# Global Transcriptome and Co-Expression Network Analysis Reveal Contrasting Response of *Japonica* and *Indica* Rice Cultivar to γ Radiation

**DOI:** 10.3390/ijms20184358

**Published:** 2019-09-05

**Authors:** Xiaoxiang Zhang, Niansheng Huang, Lanjing Mo, Minjia Lv, Yingbo Gao, Junpeng Wang, Chang Liu, Shuangyi Yin, Juan Zhou, Ning Xiao, Cunhong Pan, Yabin Xu, Guichun Dong, Zefeng Yang, Aihong Li, Jianye Huang, Yulong Wang, Youli Yao

**Affiliations:** 1 Jiangsu Key Laboratory of Crop Genetics and Physiology/Co-Innovation Center for Modern Production Technology of Grain Crops, Yangzhou University, Yangzhou 225009, China; 2 Lixiahe Agricultural Research Institute of Jiangsu Province, Yangzhou 225007, China; 3 Yangzhou Irradiation Center, Yangzhou 225007, China

**Keywords:** gamma irradiation, morphology, transcriptomic analysis, gene network, rice (*Oryza sativa* L.)

## Abstract

*Japonica* and *indica* are two important subspecies in cultivated Asian rice. Irradiation is a classical approach to induce mutations and create novel germplasm. However, little is known about the differential response between *japonica* and *indica* rice after γ radiation. Here, we utilized the RNA sequencing and Weighted Gene Co-expression Network Analysis (WGCNA) to compare the transcriptome differences between *japonica* Nipponbare (NPB) and *indica* Yangdao6 (YD6) in response to irradiation. *Japonica* subspecies are more sensitive to irradiation than the *indica* subspecies. *Indica* showed a higher seedling survival rate than *japonica*. Irradiation caused more extensive DNA damage in shoots than in roots, and the severity was higher in NPB than in YD6. GO and KEGG pathway analyses indicate that the core genes related to DNA repair and replication and cell proliferation are similarly regulated between the varieties, however the universal stress responsive genes show contrasting differential response patterns in *japonica* and *indica*. WGCNA identifies 37 co-expressing gene modules and ten candidate hub genes for each module. This provides novel evidence indicating that certain peripheral pathways may dominate the molecular networks in irradiation survival and suggests more potential target genes in breeding for universal stress tolerance in rice.

## 1. Introduction

Rice (*Oryza sativa* L.) has various nutrients with high digestibility and absorption rate, providing staple food to more than half of the world’s population, especially in Asia. Cultivated Asian rice consists of two major subspecies, *japonica* and *indica*, both are widely used in genetics, breeding, and production. The two subspecies have many different features including plant architecture, leaf color, tiller number, grain shape, nutrition quality, temperature adaptation, and cultivation region. Though many reports are focusing on their morphological, physiological, biochemical characteristics and molecular diversity [1,2,3,4], little is known about their differences in response to irradiation.

Gamma irradiation mutagenesis is an important approach to generate mutations for genetic analysis and breeding applications. Gamma ray is an ionizing radiation that penetrates and interacts with living tissue and causes DNA damage and morphological changes [5]. It enriches diversity in many aspects of the target plants, most notably in important agronomic traits like yield, quality and disease resistance [5,6]. Radiation directly acts on biologically active macromolecules of living organisms. It can alter the chemical structure of DNA, RNA and proteins, including various enzymes in cells. When the dose is high, the chemical bonds in the molecules can be broken and the metabolic processes associated with these substances might be hindered. Researchers have pointed out that gamma rays have obvious adverse effects on living organisms ranging from DNA damage to individual survival and development [7,8,9,10]. Han (2014) demonstrated that double-stranded breaks (DSBs) in genomic DNA bring the most severe results of irradiation in genomic DNA [7]. DSBs arise through the direct action of ionizing radiation, and gamma ray irradiation resulted in chromosomal aberrations, drastic mutations in human blood cells, and changes in fish larvae [11,12,13]. Gamma radiation can also act on glycolipid transfer protein, cytochrome P450, mini-chromosome maintenance family (MCM), and plant hormones to play a pivotal role in cell life cycle [14,15,16,17,18,19].

However, there is no report about the differences between *japonica* and *indica* rice subspecies in response to gamma irradiation, especially at the early germination stage. In the present study, we analyzed the differential responses of the two subspecies to irradiation, and utilized RNA sequencing and Weighted Gene Co-expression Network Analysis (WGCNA) to compare transcriptomic differences between the rice varieties Nipponbare (NPB, ssp. *japonica*) and Yangdao6 (YD6, ssp. *indica*) in response to irradiation stress at the early germination stage. Our results suggest that the mechanism in the direct DNA repair related pathway responds similarly between the subspecies, but their universal stress responses are contrastingly different. Knowledge about this difference will help us to understand the general differences of *japonica* and *indica* subspecies in withstanding radiation adversity and possibly other stressors. Furthermore, this work may offer a starting point for the elucidation of molecular networks underlying irradiation stress response in rice.

## 2. Results

### 2.1. Seedling Survival Rate Reveals Japonica Subspecies are More Sensitive to Irradiation than Its Indica Counterparts

**Figure 1:**
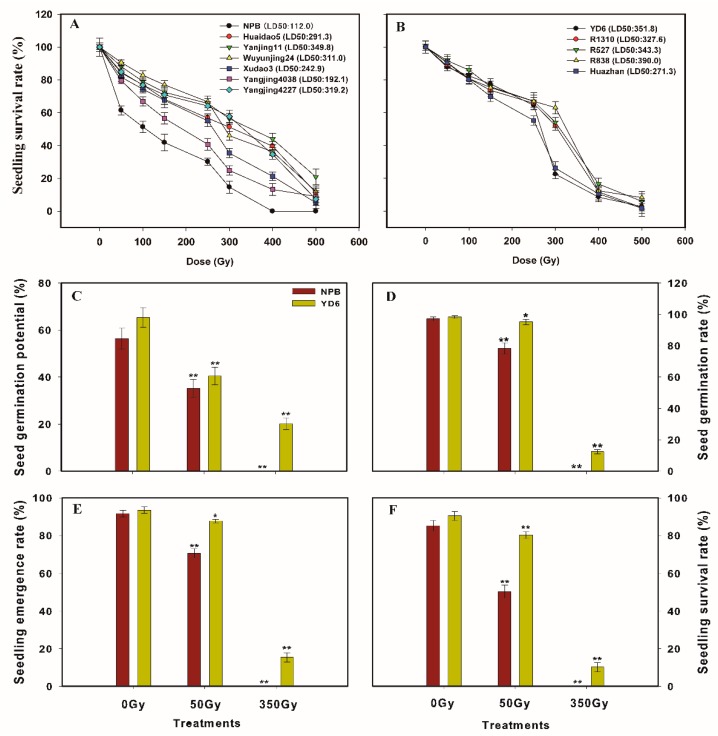
Germination profiles of *japonica* and *indica* rice at different irradiation dose. (**A**) Seedling survival rate of different *japonica* rice under different irradiation dose; (**B**) Seedling survival rate of different *indica* rice under different irradiation dose; (**C**) Seed germination potential of *japonica* rice Nipponbare (NPB) and *indica* rice Yangdao6 (YD6) under different irradiation doses; (**D**) Seed germination rate of NPB and YD6 under different irradiation dose; (**E**) Seedling emergence rate of NPB and YD6 under different irradiation dose; (**F**) Seedling survival rate of NPB and YD6 under different irradiation doses. There are three biological replicates and each biological replicate contains one hundred seeds. LD50, 50% lethal dose; Bars are mean ± SD of three independent biological replicates, and asterisks indicate significant difference (*, *p* ≤ 0.05; **, *p* ≤ 0.01) compared to control (0 Gy).

Seedling survival rate directly reflects the severity of damage triggered by irradiation of seeds. The seedling survival rates of the twelve varieties at various irradiation doses are displayed in Figure 1A,B. The *japonica* varieties generally show a faster reduction in the survival rate than that of *indica* varieties at a lower to moderate dose of irradiation, with a 50% lethal dose (LD50) ranging from 112.0 to 349.8 Gy in *japonica* (average 259.7 Gy), and 271.3 to 390.0 Gy in *indica* (average 336.8 Gy), respectively. Among them, the *japonica* rice cultivar Nipponbare (NPB) was the most vulnerable one to irradiation in the *japonica* sub-group, whereas Yangdao6 (YD6) and Huazhan were relatively the more sensitive varieties in the *indica* sub-group.

To elaborate their different germination responses to irradiation, we compared the germination potential, seed germination rate, seedling emergence rate and seedling survival rate in NPB and YD6 at three irradiation doses. All four indices were reduced significantly after irradiation (Figure 1C–F), with NPB being reduced significantly more than that of YD6. It is worthy to note that NPB showed 0.1% germination rate under an irradiation dose of 350 Gy. In contrast, the germination rate was about 12.4% in YD6 and survival rate remained to be 10.1% at the same dose. This further confirms that NPB was more susceptible to irradiation than YD6.

### 2.2. Irradiation Imposes More Inhibition on Root Growth than Shoot, More in NPB than in YD6

After subjected dry seeds to irradiation, the length of both shoot and root in germinating seedling was significantly reduced in all varieties (Figures S1–S3). Shoot length and root length displayed an exponential relationship, regardless of variety. Irradiation triggered more pronounced reduction in root length than in shoot length. This indicates that although the growth of shoot and root are coordinated under normal condition, irradiation imposed a greater inhibition to the extension of root rather than shoot.

**Figure 2:**
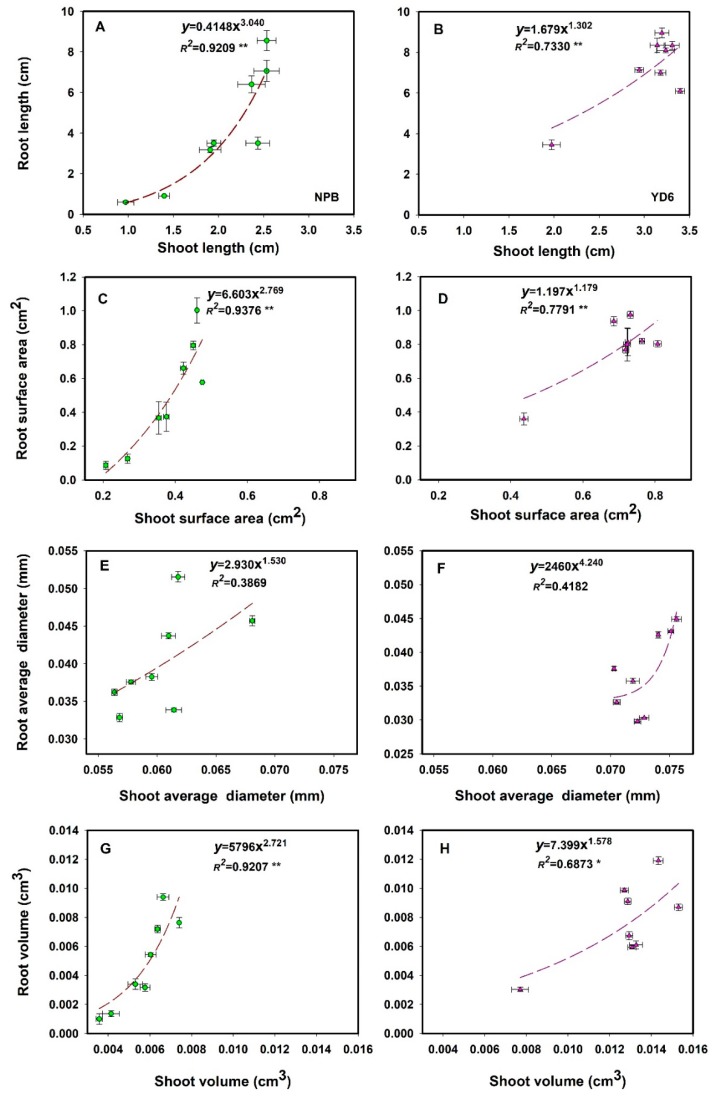
Correlation between four indices of shoot and root at different irradiation dose (0 Gy, 50 Gy, 100 Gy, 150 Gy, 250 Gy, 300 Gy, 400 Gy and 500 Gy) in *japonica* rice NPB and *indica* rice YD6. Three biological replicates and each biological replicate contains one hundred seeds. * (*p* ≤ 0.05), ** (*p* ≤ 0.01) represent significance level.

To define the varietal differences in the shoot and root features, we explored the relationships among length, surface area, average diameter and volume of shoot and root. The determination coefficient (*R*^2^ value) of these features were all significant (*p* *≤* 0.01), with root length, root surface area and root volume showing more inhibited in NPB than in YD6 (Figure 2). This suggests that probably these indices explained morphological why YD6 was more tolerant to irradiation than NPB. Therefore, in most of the following analysis, we used NPB and YD6 to represent the sensitive and tolerant rice varieties, respectively.

### 2.3. More Extensive DNA Damage Incurred in NPB than in YD6 Even under a Low Dose Irradiation

**Figure 3:**
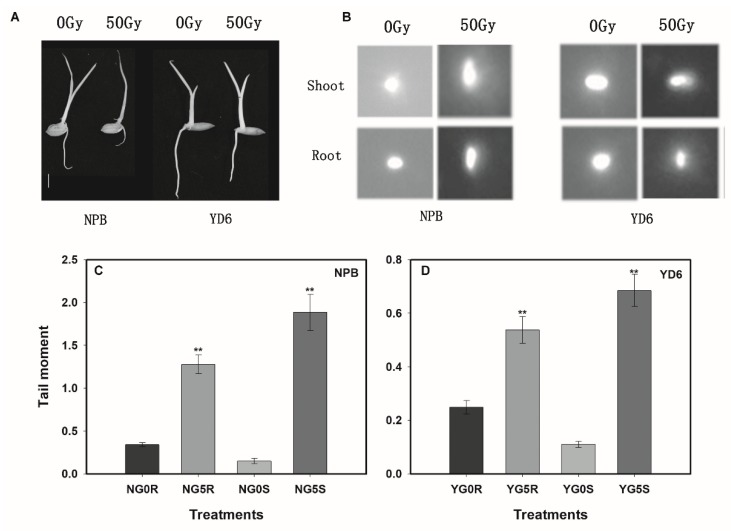
Comet assay of shoot and root cells. (**A**) Morphological characteristics of seedlings of NPB and YD6. Bar = 1 cm; (**B**) nuclei collected from shoot and root of NPB and YD6 treated with 0 Gy and 50 Gy; (**C**) the tail moment values of NPB. NG0R and NG0S, the control (0 Gy) of root and shoot in NPB, respectively. NG5R and NG5S, the irradiation treatment (50 Gy) of root and shoot in NPB, respectively; (**D**) the tail moment values of YD6. YG0R and YG0S, the control of root and shoot in YD6, respectively. YG5R and YG5S, the irradiation treatment of root and shoot in YD6, respectively. ** (*p* ≤ 0.01) indicates significant difference compared to control.

To elucidate the possible differences in DNA damages between NPB and YD6, we ran comet assay and compared the tail moment of shoot and root tissue induced by a dose of 50 Gy. Obviously, irradiation inhibited the root length more than that of the shoot, and NPB showed a more significant reduction in the length than that of YD6 (Figure 3A). Comet assay reveals that the nucleic DNA fragments migrated more extensively in NPB than that in YD6, irrespective of tissues (shoot or root, Figure 3B). The tail moment value, which represents the extent of DNA damage, was significantly higher in NPB than that in YD6 (Figure 3C,D). However, the tail moment values were higher in shoot rather than root, regardless of variety. These results indicate that irradiation caused DNA damage in both shoot and root tissues, and the degree of DNA damage was more extensive in shoot than in root, more in NPB than in YD6. Though the comet assay result was consistent with the varietal differences regarding their vulnerability to irradiation, however, it is obscured by different morphological responses of shoot and root tissues.

### 2.4. RNA Sequencing Analysis on Shoot and Root of NPB and YD6

To investigate transcriptional differences in response to the irradiation, RNA-sequencing was employed to explore transcriptomic changes in shoot and root tissues regarding mock (0 Gy) and 50 Gy treatments of NPB and YD6. An average 47.4 million clean reads (range 38.7–55.0) were generated in each sample, corresponding to a total of 7.1G bp clean bases (range 5.8–8.2) indicating evenly distributed sequencing depth across treatments (Table S1). The proportion of high-quality base was 95.4%, with a Q30 at 92.1%. Principal component analysis (PCA) revealed that samples of NPB are more closely clustered while samples of YD6 being more sparsely distributed (Figure S4A). Regardless of variety, root samples were distributed distantly from shoot samples and irradiation treatment lead to more sparsely distribution in YD6 than in NPB. Correlation and clustering analysis reveal that samples of biological replicates are highly reproducible and consistent, while tissue and variety introduce scaled up differences (Figure S4B). All these indicate that the sequencing reads are highly reliable.

The high-quality clean reads were assembled by the Cufflinks algorithm [20]. The reads mapped 94.61% to the coding sequence (CDS) and 3.05% to the introns in NPB (Figure S5A,B). Those numbers were 95.56% and 2.16% in YD6, respectively (Figure S5C,D). These results indicate that the clean reads distribution was consistent between the two varieties, suggesting that the expression pattern in gene locations is basically homogenous between the varieties.

### 2.5. Validation of RNA-Seq Data by Quantitative Real-Time PCR (qRT-PCR)

**Figure 4:**
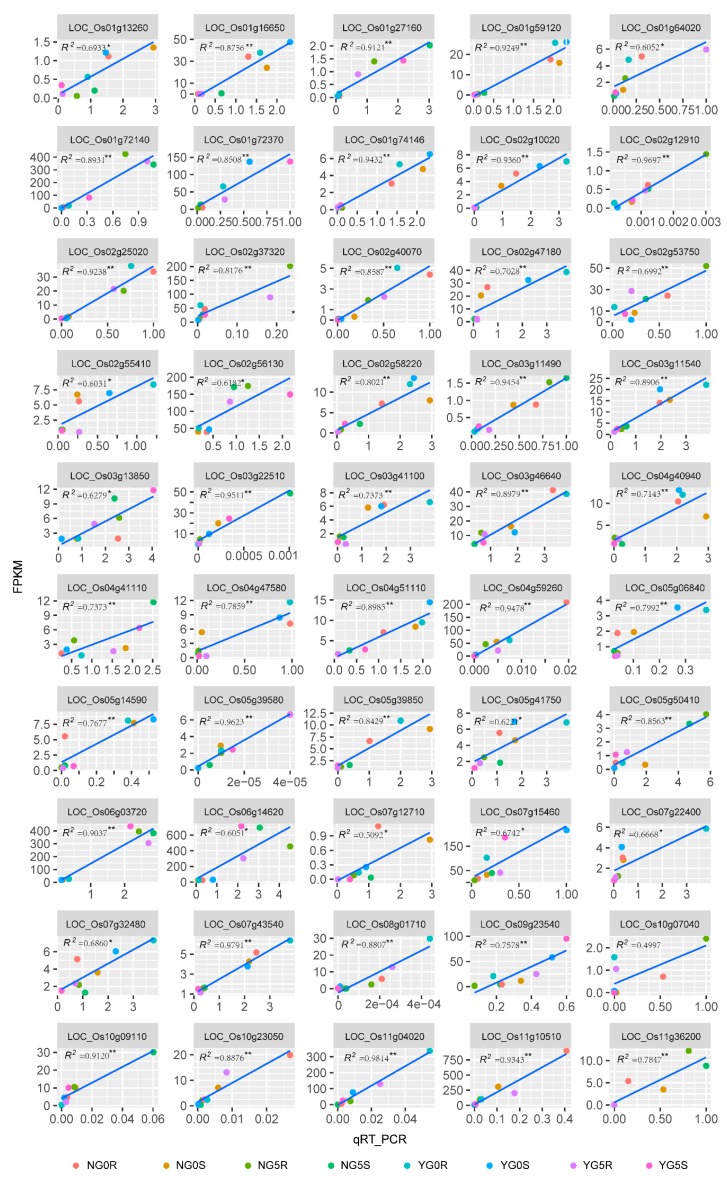
Correlation of RNA-seq results and qRT-PCR validation in 50 representative genes. * (*p* ≤ 0.05) and ** (*p* ≤ 0.01) represents significant difference.

To validate the RNA sequencing data, we selected 50 genes (41 DEGs and 9 hubgenes) (Table S2), which represent different FPKM levels, and detected their expression levels by qRT-PCR from the same batch of RNA samples for sequencing. The determination coefficient (*R*^2^ value) of the qRT-PCR validation data against the FPKM data was positively significant at *p* ≤ 0.05 in 49 of the 50 genes (ranging from 0.6021 to 0.9814; Figure 4), except one was significant at *p* ≤ 0.10 (*R*^2^ = 0.4997). The consistency between the qRT-PCR validation and the RNA sequencing results confirms the reliability of the RNA-Seq analysis.

### 2.6. Gene Ontology (GO) Pathway and MapMan Analysis of DEGs

**Figure 5:**
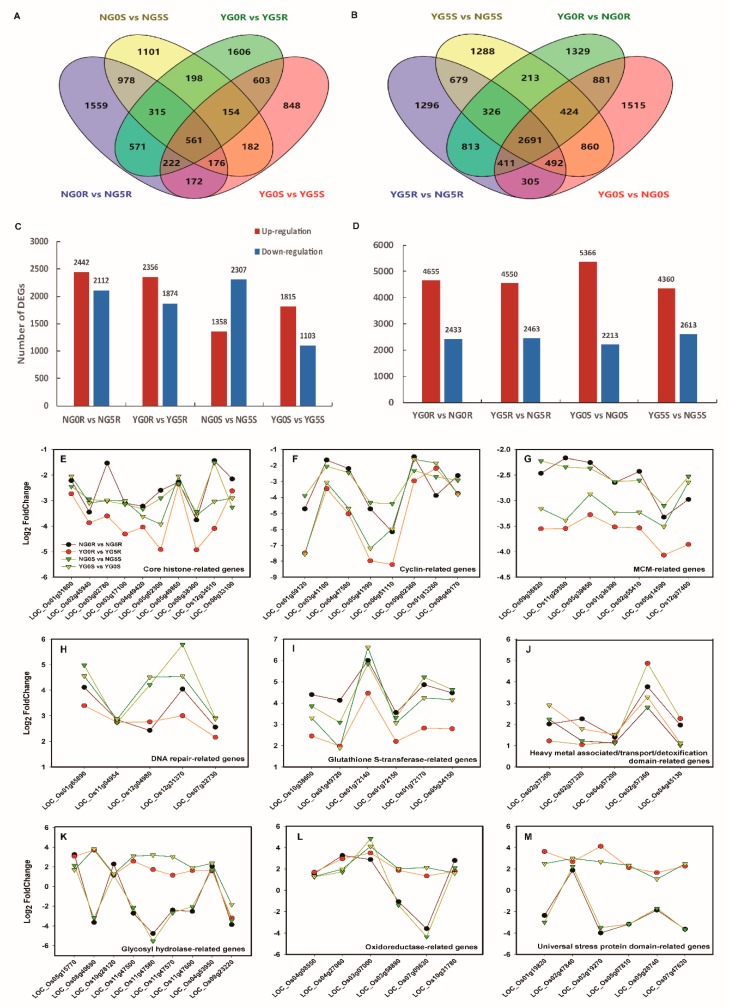
Differences of global gene expression profiling in shoot and root of NPB and YD6 under irradiation. (**A**,**B**) Venn diagrams of differentially expressed genes (DEGs) between irradiation response (**A**) and varietal effect (**B**); (**C**,**D**) Numbers of DEGs in each comparison between irradiation response (**C**) and varietal effect (**D**); (**E**–**M**) Changes of some DEGs associated with core histone (**E**), cyclin (**F**), MCM (**G**), DNA-repair (**H**), glutathione S-transferase (**I**), heavy metal associated/transport/detoxification (**J**), glycosyl hydrolase (**K**), oxidoreductase (**L**) and universal stress protein (**M**) in response to irradiation.

The numbers of differentially expressed genes (DEG) are shown in Figure 5A–D. The total numbers of DEG in response to the irradiation in NPB were 4554 and 3665 for root and shoot tissues, respectively. Those DEGs in YD6 were 4230 and 2918, respectively. The DEGs in NPB were 7.7% and 25.6% more than those in YD6, for root and shoot, respectively. The common DEGs in response to the irradiation were 1669 and 1073 in root and shoot, respectively. Among them, the common DEGs between root and shoot were 561 only (Table S3, Figure S6A,C), with the majority being exclusive DEGs, suggesting contrasting different responses to irradiation between different tissues. The unique DEGs in the root were 2885 and 2561 for NPB and YD6, respectively; correspondingly, the unique DEGs in the shoot were 2592 and 1845, respectively. Similar to the total DEGs, the unique DEGs were 12.6% and 40.5% more in NPB than in YD6 for root and shoot, respectively. These indicate that the irradiation triggered more significant changes in the root than in the shoot, and more drastic transcriptional changes in the irradiation vulnerable variety NPB than in the irradiation tolerant variety YD6.

The varietal DEGs directly reflect the genetic differences between the two varieties. Regardless of tissues and treatment groups, there were 2691 common DEGs in varietal comparison groups (Figure 5B, Table S4, Figure S6B,D), which was 19.9% of the total DEGs. This number dwarfs the irradiation responsive common DEGs (561, 6.1% of its total DEGs). Of these two groups of common DEGs, only 43 genes overlapped. These indicate that intrinsic varietal differences overwrite the scale of irradiation effect.

To further inquire the functions and pathways of irradiation responsive common 561 DEGs involved in, we calculated their transcription level changes and classified them into three categories: induced in root and shoot of both varieties (207, 36.9%; Table S5), suppressed in both varieties (236, 42.1%; Table S6), and induced in one variety but suppressed in the other (82, 14.6%; Figure S7 and Table S7). Gene ontology (GO) annotation analysis reveals that the biological process of the commonly induced DEGs fall into DNA repair, response to DNA damage stimulus, regulation of nucleobase, nucleoside, nucleotide and nucleic acid metabolic process, regulation of cellular biosynthetic process, regulation of nitrogen compound metabolic process, regulation of macromolecule biosynthetic and metabolic processes, regulation of transcription and gene expression etc. The molecular function of the induced DEGs are hydrolase activity, DNA-dependent ATPase activity, nucleoside-triphosphatase activity, pyrophosphatase activity, ATPase activity and helicase activity. All these point to that DNA repair related processes are actively up-regulated.

In contrast, the commonly suppressed DEGs fall into the biological process of DNA conformation change, DNA replication initiation, DNA-dependent DNA replication, DNA packaging, nucleosome organization, nucleosome and chromatin assembly, protein-DNA complex assembly, cellular macromolecular subunit organization and complex assembly, microtubule-based process and movement, cellular component organization, biogenesis and assembly, chromatin and chromosome organization, organelle organization and cellular process. Their molecular functions are microtubule motor activity, pyrophosphatase activity, purine ribonucleotide binding, ATP binding, adenyl ribonucleotide binding and protein serine/threonine kinase activity. It is worth mentioning that all eight genes of the DNA replication initiation pathway, and 11 of 17 DNA-dependent DNA replication genes were down-regulated, indicating DNA replication is very inactive upon irradiation.

Of those 82 common DEGs of contrasting expression profiles in YD6 and NYB (Table S7), the biological process they fall into are oxidation reduction, responses to stress and stimulus, and carbohydrate metabolic process. Their molecular functions are oxidoreductase activity, glycosyl hydrolase activity, and cation, ion (including iron ion), heme and tetrapyrrole binding. Assumingly, these differences contribute critically to their differential responses to irradiation between the two varieties.

**Figure 6:**
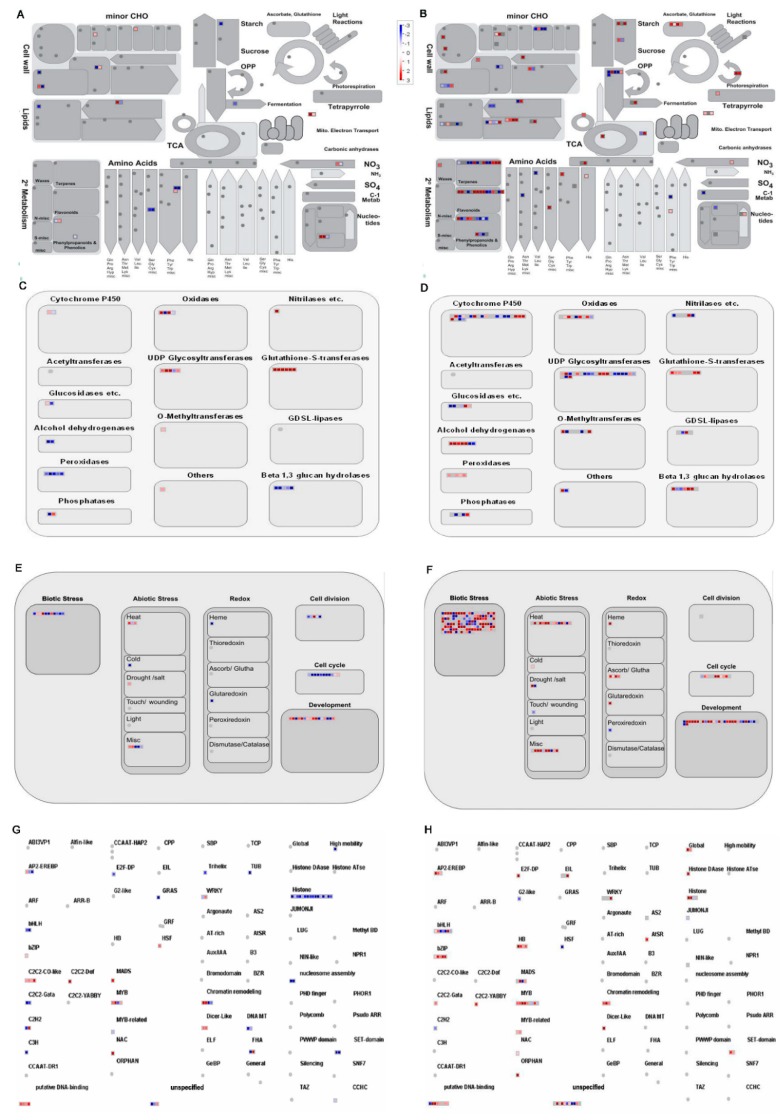
MapMan analysis of DEGs in irradiation response (**A**,**C**,**E**,**G**) and cultivar effect (**B**,**D**,**F**,**H**). Results of mapping 552 and 2350 genes to metabolism (**A**,**B**), large enzyme families (**C**,**D**), cellular response (**E**,**F**) and transcriptions (**G**,**H**). Red boxes, up-regulated genes; blue boxes, down-regulated genes.

MapMan analysis was used to further identify the related metabolic pathways in irradiation response (Figure 6). We found the pathway for nucleotides metabolism (Figure 6A), oxidases, uridine diphosphate (UDP) glycosyltransferases and glutathione-S-transferases (Figure 6C), development (Figure 6E), and putative DNA binding (Figure 6G) were the most significantly up-regulated pathway in response to irradiation. In contrast, amino acids in metabolism, peroxidases in large enzyme families, cell cycle in cellular response, histone in transcriptions factors were the most significantly down-regulated pathway. This suggests that these differences in metabolism pathways are critical to the differential responses to irradiation.

Of those common 2691 DEGs between the varieties, we specifically searched for those that showed opposite changes in response to irradiation in YD6 and NYB (Tables S8–S10). In total, 47 genes were found falling to this category (Figure S8). Among them, six genes (LOC_Os01g73200, LOC_Os02g49720, LOC_Os08g36480, LOC_Os09g32570, LOC_Os10g02070, LOC_Os11g10510) were enriched to the biological process of oxidation reduction (GO:0055114). This suggests that differential response in oxidation reduction between the two varieties plays a significant role to their differential response to irradiation.

It is worth specifically mentioning that, among those common irradiation responsive DEGs, we found that most of the core histone H2A/H2B/H3/H4 domains containing protein coding genes were down-regulated upon irradiation (Figure 5E). Similarly, eight cyclin related genes and seven minichromosome maintenance genes (MCM) were also significantly reduced after irradiation (Figure 5F,G). These results imply that these genes were coordinately suppressed in response to irradiation, leading to inhibited DNA replication and slow-down cell proliferation. In addition, DNA repair related genes were induced (Figure 5H), suggesting more activation of DNA repairing process; multiple stresses responsive genes such as glutathione S-transferase (Figure 5I), heavy-metal detoxification domain containing genes (Figure 5J), glycosyl-hydrolase (Figure 5K), oxidoreductase (Figure 5L) and universal stress protein domain containing genes (Figure 5M) were significantly altered in response to irradiation, suggesting various stress genes participate in post irradiation recovery process.

Similarly, among those varietal DEGs, we found that AP2 domain, B3 DNA binding domain, calmodulin binding protein, glutathione S-transferase, heavy metal associated/transport/detoxification, NBS-LRR disease resistance protein, cytochrome P450, glycosyl hydrolase, transponson protein, UDP-glucoronosyl/UDP-glucosyl transferase, zinc finger protein and leucine rich repeat protein related genes (Figure S9A–L) were significantly altered differently, suggesting that some of these genes defined the varietal differences.

Meanwhile, MapMan analysis was used to further identify the important metabolic pathways in varietal response (Figure 6). We found that the pathway for TCA and OPP in metabolism (Figure 6B), oxidases, cytochrome P450, and glutathione-S-transferases in large enzyme families (Figure 6D), biotic stress and development in cellular response (Figure 6F), bZIP, HB, MYB and HSF in transcriptions factors (Figure 6H) were the most significantly altered pathways in varietal differences. This suggests that these metabolism pathways defined the varietal differences.

Additionally, we also analyzed the DEGs between root and shoot, i.e., the tissue effect. There were 3673 common DEGs in tissue comparison groups (Figure S10A, Tables S11 and S12). The tissue DEGs reflect the tissue specific regulation. Gene ontology (GO) annotation analysis reveals that the biological processes of the DEGs fall into oxidation reduction, regulation of nitrogen compound metabolic process, regulation of transcription, regulation of nucleobase, nucleoside, nucleotide, and nucleic acid metabolic process, regulation of biosynthetic process etc. The molecular functions of the DEGs are oxidoreductase activity, iron ion binding, heme binding, tetrapyrrole binding etc. The cellular components of the DEGs include membrane, extracellular region, thylakoid, photosystem, photosynthetic membrane (Figure S10B, Table S12). MapMan analysis reveals that the pathway for the cell wall and OPP in metabolism (Figure S10C), development, cell cycle and cell division in cellular response (Figure S10D), cytochrome P450, oxidases, and glutathione-S-transferases in large enzyme families (Figure S10E), HB, WRKY, MYB and bHLH in transcriptions factors (Figure S10F) were the most significant altered pathways in tissue differences. This suggests that oxidation reduction, as well as metabolic differences contributed to the differential response to irradiation even under the same genetic background between root and shoot tissues.

### 2.7. Weighted Gene Co-Expression Network Analysis (WGCNA) Identifies Candidate Pivotal Modules Associated with Seedling Growth Traits

**Figure 7:**
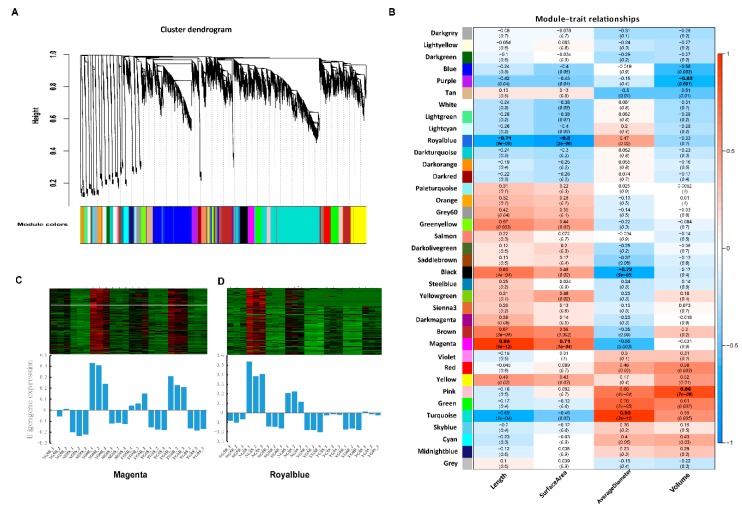
Weighted gene co-expression network analysis (WGCNA) of gene expressions and traits. (**A**) Hierarchical cluster tree showing 37 co-expression modules based on WGCNA. Each branch in the tree represents an individual gene; (**B**) Module—trait relationships and corresponding *p*-value. The panel on the left shows 37 modules and the panel on the right is the relevant color scale from −1 to 1. The numbers above the parentheses represent the correlation coefficient (*r*), and the numbers in parentheses represent the significance (*p*). (**C**) Top half is heatmap showing the FPKM and lower part is the eigengene expression profile in magenta module; (**D**) Top half is heatmap showing the FPKM of each gene and lower part is the eigengene expression profile in royal blue module.

To identify candidate pivotal genes associated with seedling growth traits, WGCNA [21] was performed to classify specific genes that are associated with the phenotype scale including root length, surface area, average diameter, and volume. After filtering out the genes with a low FPKM level, 27074 genes were retained for the subsequent WGCNAs. This analysis identified thirty-seven distinct co-expression modules (labeled with diverse colors) shown in the dendrogram (Figure 7A), of which nine modules (magenta, turquoise, pink, royal blue, black, brown, green yellow, purple and blue) showed a significant association with the four different phenotype traits (Figure 7B). Figure 7C,D show the eigengene expression for magenta and royal blue module, respectively. Other expression data from different module are shown in Figure S11. The scale of length and surface area were highly positively correlated with the gene expression in the magenta module, with correlation coefficient (*r*) at 0.94 (*p* = 6 × 10^−12^) and 0.71 (*p* = 1 × 10^−4^), respectively; but these traits were negatively correlated with the royal blue module. The scale of average diameter showed a positive correlation with the turquoise module (*r* = 0.93, *p* = 3 × 10^−11^), but negatively correlated with the value of the royal blue module (*r* = −0.72, *p* = 9 × 10^−5^). Finally, the size of this volume was in high positively correlation with the pink module (*r* = 0.86, *p* = 7 × 10^−8^) and negatively correlated with the purple module (*r* = −0.63, *p* = 0.001). GO enrichment analysis for these selected modules are shown in Tables S13–S18. These reveal that the phenotypic traits are more closely related to certain modules of genes than others.

### 2.8. Construction of the Gene Networks and Establishment of Candidate Hub Genes

**Figure 8:**
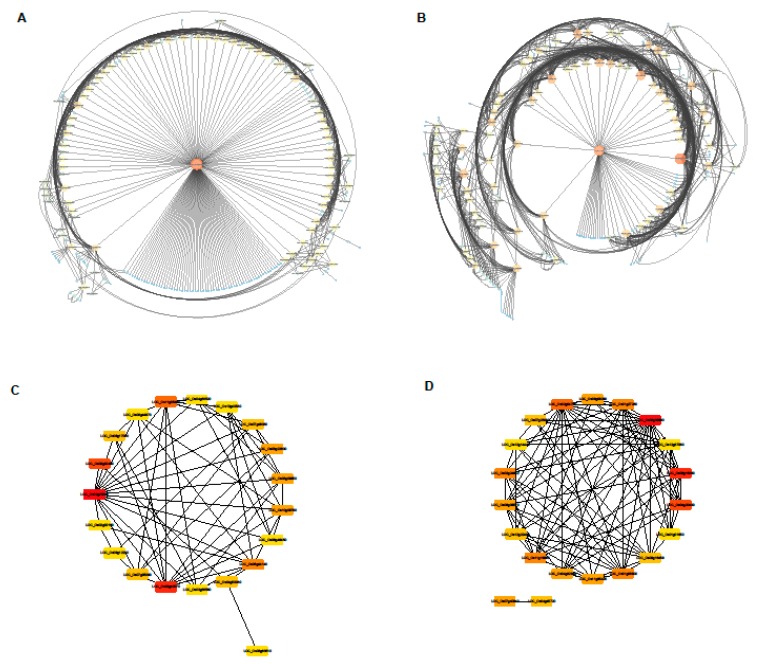
Co-expression network analysis of length and surface area related modules. (**A**,**B**) Gene co-expression networks of positive correlation magenta module (**A**) and negative correlation royal blue module (**B**) visualized using Cytoscape software platform. The size of the circle and the depth of the color indicate the degree of connectivity of the gene; (**C**,**D**) The correlation networks of top 20 nodes in magenta module (**C**) and royal blue module (**D**). The depth of the color represents the number of associated nodes, respectively.

To construct the gene networks, the highest significant correlated modules (one each from positively and negatively correlated modules) were selected. Since the scale of length and diameter share the same selected modules, totally six most relevant modules associated with the four phenotypic traits were chosen for the analysis. The co-expression network diagrams were generated basing on the degree of association of the genes within the module (Figure 8A,B, Figures S12A,B and S13A,B). The candidate hub genes are screened by their closest connections through the KME value (eigengene connectivity) in gene network. The top 20 node genes from each module were selected to generate a network map (Figure 8C,D, Figures S12C,D and S13C,D).

To screen for candidate hub genes, the top ten connected genes with their KME value in the specific modules are shown in Table 1, Table 2 and Table 3. The scale of length and surface area is related positively to the magenta module and negatively to the royal blue module, respectively (Table 1). In the magenta module, the top ten genes encode transcription factors and other proteins, including auxin-induced protein, AT hook motif domain containing protein, CPuORF8—conserved peptide uORF-containing transcript, ATPase BadF/BadG/BcrA/BcrD type, LTPL146—protease inhibitor/seed storage/LTP family protein precursor, DNA binding protein, peroxidase precursor, and AP2-like ethylene-responsive transcription factor PLETHORA 2. The correlation network of the magenta module is shown in Figure 8A. Genes encoding the aforementioned proteins were identified as key candidate hub genes for the magenta module (Figure 8C). In the royal blue module, the top ten genes encode transferase and other proteins, including O-methyltransferase, aldehyde dehydrogenase, glutathione S-transferase, sulfotransferase, flavonol synthase/flavanone 3-hydroxylase, photosystem II reaction center protein K precursor, transposon protein, and expressed protein. The correlation network of the royal blue module is shown in Figure 8B. Genes encoding these proteins were identified as key candidate hub genes for the royal blue module (Figure 8D). Multiple transferases were enriched in this module, indicating that transferase regulatory network may play a pivotal role in regulation of the scale of length and surface area.

The scale of average diameter is related positively to the turquoise module and negatively to the black module, respectively (Table 2). In the turquoise module, the top ten genes encode proteases and other proteins, including peroxidase, aminotransferase, chalcone synthase, peroxidase precursor, BBTI6—Bowman-Birk type bran trypsin inhibitor precursor, leucine-rich repeat receptor protein kinase EXS precursor, fimbrin-like protein 2 and carrier. The correlation network of the turquoise module is shown in Figure S12A. In the black module, the top ten genes encode transcription factors and other proteins, including transcription factor (WRKY11 and WRKY107), receptor-like protein kinase like protein, cytochrome P450 72A1, UP-9A, basic 7S globulin 2 precursor, hydrolase, UDP-glucoronosyl and UDP-glucosyl transferase domain containing protein, and heavy metal-associated domain containing protein (Table 2 and Figure S12C). The correlation network of the black module is shown in Figure S12B,D. Several transcription factors were enriched in this module, indicating that transcription factors may play a crucial role in regulation of the scale of average diameter.

The scale of volume is related positively to the pink module and negatively to purple module (Table 3). In the pink module, the top ten genes contain tRNA-splicing endonuclease positive effectors, chalcone and stilbene synthases, peptide transporter, metal transporter Nramp6, cytochrome b559 subunit alpha, dehydrogenase, ZOS8-05-C2H2 zinc finger protein, helix-loop-helix DNA-binding domain containing protein and retrotransposon protein. The correlation network of the pink module is shown in Figure S13A,C. In the purple module, the top ten genes contain phytosulfokine receptor precursor, transposon protein, receptor-like protein kinase 2 precursor, lysM domain containing protein, and MYB family transcription factor. The correlation network of the purple module is shown in Figure S13B,D.

Interestingly, we find 21 hubgenes are DEGs in this study (Table 1, Table 2 and Table 3). The hubgenes are at the nodes of the gene expression network, which may be located upstream of certain pathways, and changes of hubgenes mean that other genes will also change accordingly.

**Figure 9:**
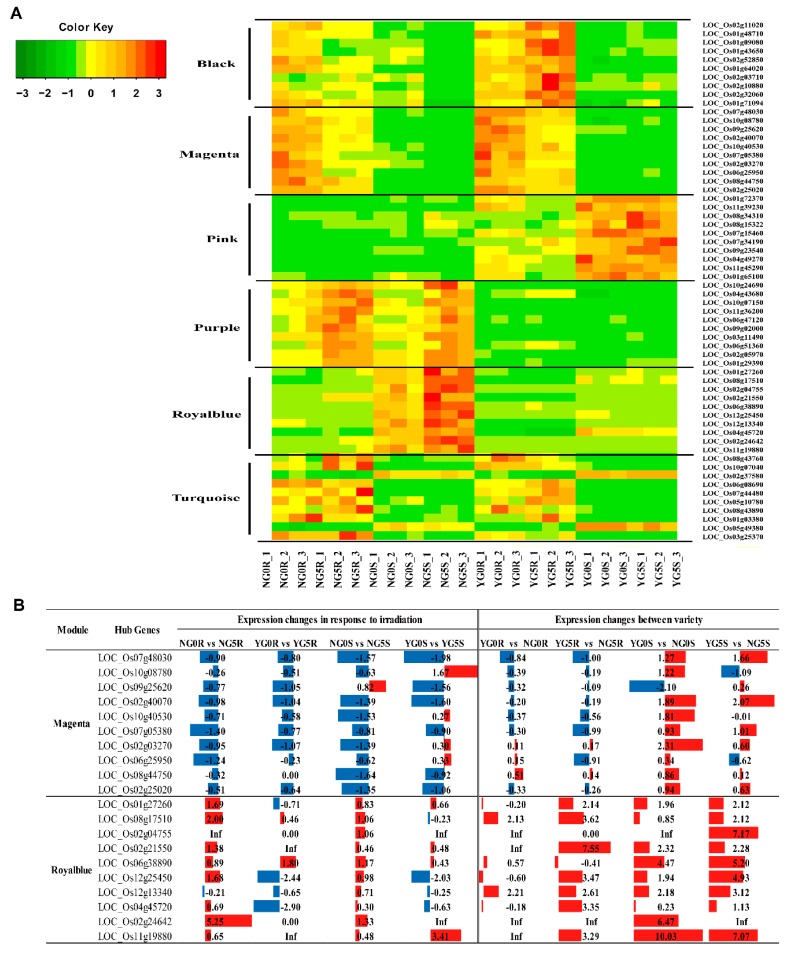
Gene expressions of top ten node genes of six related modules. (**A**) Heatmap comparison showing the expression profiles of top ten node genes in each module; (**B**) Changes in the expression levels of hub genes in top ten node genes of magenta module and royal blue module.

The heatmap of the candidate hub genes from different modules shows that the response of genes subject to the variety and tissue (Figure 9A). In the black and magenta modules, the expression level of the most genes display a suppression in the roots of NPB, whereas induction is observed in YD6. These indicate that the top ten genes are more active in the roots between NPB and YD6 in the black and magenta module. However, these genes are more active in the shoot and root of NPB than YD6 in the purple and royal blue modules. Interestingly, the genes in the pink module are the most active in the shoot of YD6 only. These contrasting differences may contribute to their differential tolerance to the irradiation as well. Furthermore, the expression levels of these module genes are up- or down-regulated in the irradiation response and between the varieties (Figure 9B and Figure S14). These important genes enriched in different modules indicate that hub genes may play a crucial role in the differential responses to irradiation of NPB and YD6.

## 3. Discussion

*Japonica* and *indica* rice are two important subspecies in the cultivated Asian rice group for rice production, especially in Asian regions. Recent reports have renewed our understanding of their divergence, indicating that they are domesticated independently, with limited intergroup introgression [2,22]. This diversification defines many of their differences in morphological as well as agronomical traits. However, their possible difference in response to radiation is almost unknown, especially in the transcriptomic aspects. In rice production practice, irradiation mutagenesis can introduce new mutations, which are important for the innovation of germplasm and widely used in breeding applications. In our study, when dry seeds are subjected to irradiation, *indica* showed a higher seedling survival rate than *japonica*. The molecular mechanism underlying the differential responses is lacking.

It has been demonstrated that irradiation has different comprehensive effects on the physical and chemical properties, besides structural properties of starch, the amylose content, carboxyl content and acidity increase after irradiation [23]. After ion irradiation in rice, the growth retardation and death of rice are related to early dose and time-related gene expression changes [24]. In wheat, gamma irradiation can stimulate increase in the number of wheat seedlings with various developmental disorders and wheat seed progeny also had a lower seedling survival rate and weight [25]. In other plants, it has been demonstrated that plants alter the contents of a range of primary metabolites, such as carbohydrates, organic acids, and amino acids in response to UV-B radiation [26,27,28]. In this study, the *japonica* rice was found to be more sensitive to radiation in terms of seed germination potential, seed germination rate, seedling emergence rate and seedling survival rate. *Indica* rice shows better tolerance and therefore has a higher seedling survival rate than *japonica* rice after irradiation. The effects of irradiation on the morphology of roots and shoots are different, and the root elongation is more affected. These indicates that the effect of irradiation on *japonica* from phenotype to DNA level is more serious than that of *indica*. Even at low dose range, we found irradiation caused more extensive DNA damage in *japonica* rice than to *indica* rice. Studies in onion have shown that main contribution to the overall frequency of chromosomal aberrations is made by the bridge-type aberrations, fragments, and lagging chromosomes after irradiation, in addition to a variety of aberrations [29]. What type of DNA damage differences between *japonica* and *indica* subspecies is still waiting for clarification?

Gamma radiation induces a wide range of damage and the irradiated cells must counteract these damages by activating complex reaction pathways [30]. Leaf growth is closely associated with photosynthesis, indicating that the light-dependent response and growth reduction by UV-B radiation is remarkable during the photosynthetic period [31,32,33,34]. In this study, GO analysis in molecular function were enriched in DNA metabolic process, cell cycle, cellular component organization in responsive to irradiation, etc., (Figure S6A). KEGG pathway analysis revealed that most DEGs were categorized into cell cycle, DNA replication, etc., (Figure S6C). DNA repair proteins related genes were influenced by irradiation stress. Homologous recombination (HR) is a significant biological activity in the nucleus, indispensable for the maintenance of genome consistency, integrity, stability and closely relative to the plant and animal survival. In most cases, HR during meiosis resulted in the exchange of homologous alleles from the two parents, which enhanced the genetic diversity of the offspring. At the same time, it also guarantees the proper separation of homologous chromosomes. In the mitosis of somatic cells, homologous recombination enables DNA double-strand breaks (DSBs) and cross-linked DNA and damaged replication forks to be repaired to ensure DNA replication [35,36]. In our study, DNA repair protein Rad51 which binding to ssDNA, homologous DNA pairing, strand invasion and exchange. RAD51, an eukaryote gene which is a member of the RAD51 protein family, assist in repair of DNA DSBs [37,38]. DSBs repair by homologous recombination is initiated by 5′ to 3′ strand resection (DSB resection). Rad51 has a pivotal function in meiotic prophase in mice and its loss leads to depletion of late prophase I spermatocytes [39]. During meiosis, the two recombinases, Rad51 and Dmc1, interact with single-stranded DNA to form specialized filaments that are adapted for facilitating recombination between homologous chromosomes [40]. Both Rad51 and Dmc1 have an intrinsic ability to self-aggregate. In present study, we found the changes of Rad21/Rec8, Rad15 and Rad17 were up-regulated associated with irradiation. On the other hand, genes associated with MCM were up-regulated, core histone-associated genes were down-regulated, and cyclin related genes were down-regulated (Figure 5E–M). The core genes related to DNA repair and replication and cell proliferation are similarly regulated by irradiation, however the universal stress responsive genes are in contrasting differential response pattern between the *japonica* and indicia rice subspecies. Tissue effect DEG analysis also corroborate the above view that oxidation reduction as well as metabolic differences may define a differential irradiation response, as we have seen in the contrasting root and shoot vulnerability to radiation.

Similarly, we found groups of genes that displayed opposite regulation in the two varieties, either in irradiation response (Figure S7) or between the varieties (Figure S8). They are very likely defining the differential response of NPB and YD6 to irradiation. The regulatory pathways of these genes ultimately affect NPB and YD6 in phenotype and physiological differences.

In addition, we found that expressions of *SHORT INTERNODES* (*OsSIN*, LOC_Os03g22510) were all up-regulated in all combinations. The *OsSIN* gene encodes a small protein located in the nucleus [41]. Its overexpression leads to shorter internodes, dwarf height and reduced number of inferior grains [41]. In our study, compared to root, the expression of shoot of *OsSIN* is more than 2-fold in irradiation effect (Table S3). In cultivar effect, the fold change of shoot compared to root is more than 31-fold in YD6 (Table S4). However, the fold change of shoot is only more than 9-fold in NPB. This may indicate that the response of *OsSIN* gene involved in the irradiation response is more active in *indica* than *japonica*.

Another new discovery in this study is that we identified modules related to phenotype traits. WGCNAs serve as gene modules for finding synergistic expression and can explore the relationship between gene networks and phenotypes. We found six key modules associated with the scale of length, surface area, average diameter and volume. In the gene network, we found some important hormone, transcription factors, transferase proteins, DNA binding protein, and carrier proteins appear in the hub genes.

The AP2/ERF family has a highly conserved DNA binding domain called the AP2 domain and is only present in the plant kingdom. AP2/ERF genes are from a large multigene family and play important roles through the cycle of the plant life, including the respond to various types of biotic and abiotic stress [42]. In this study, we found a hub gene (LOC_Os02g40070) associated with AP2-like ethylene-responsive transcription factor PLETHORA 2 (Figure 8A,C and Table 1). The difference in the expression level of this gene is mainly reflected in the shoot site. There was a difference between YD6 and NPB in shoot before irradiation, but there was no difference after irradiation. This indicates that the expression level of the transcription factor in *indica* increased significantly after irradiation, reaching the expression level in *japonica*, thereby regulating the expression of downstream genes in co-expression network. These results led to differences in root length and shoot length between *indica* and *japonica* rice. The final performance is the difference in emergence rate and seedling rate.

AT-hook protein has important regulatory effects on plant growth and development. The AT-hook is a DNA-binding motif present in many proteins, including the high mobility group (HMG) proteins, DNA-binding proteins from plants and hBRG1 protein, a central ATPase of the humans witching/sucrose non-fermenting (SWI/SNF) remodeling complex [3]. In this study, we found the expression level of AT-hook protein (LOC_Os02g03270) showed a great difference in the shoots of *indica* and *japonica* rice, and the change was significant after irradiation (Figure 8B and Table 1). All these hub genes work together to regulate the growth and development of root and shoot length, thus affecting the growth difference of *indica* and *japonica*.

Transcription factors are thought to play a key role in the activation and fine-tuning of various stress defense responses in plants [43]. WRKY gene family is a plant-specific zinc finger type transcription factor that is widely involved in a variety of abiotic stress response [44]. In most previous studies, most of the WRKY genes are related to the disease resistance and other stressors of plants. In Arabidopsis thaliana, the transcription factor WRKY11 acts as a negative regulator of basal resistance to *Pst* [43]. In this study, we found two WRKY11 and WRKY107 (LOC_Os01g43650 and LOC_Os01g09080) are hub genes in regulation networks of black module (Table 2) and WRKY107 also belongs to the DEGs. This indicates WRKY genes involvement in plant growth and development by regulating gene expression. Then through various networks of certain pathways to regulate the expression of other genes to achieve its biological significance in plants.

The major transcriptional reprogramming associated with the plant defense response requires not only different transcription factors but also the action of plant hormones [45,46]. As an important plant hormone, auxin affects the growth and development of plants by regulating the synthesis of RNA and proteins. In this study, we found that the response of an auxin-induced protein 5NG4 (LOC_Os08g44750) to irradiation is obvious in shoots of *japonica*.

These differences are the results of joint regulation of many genes. We found the hub genes in so many different genes through the co-expression network, and found that these hub genes have a high degree of connectivity with other key genes. Changes in the level of core gene expression mean that many other genes also change. We also found that the expression patterns of these core genes were different before and after irradiation among cultivars. The difference in these expression patterns led to the difference in the response of rice to irradiation, and the difference in germination potential, germination rate, emergence rate, and seedling rate between *indica* and *japonica*.

Yet, transcriptomic dissection like this work shows can only reveal an integrative coordinated general differential response to irradiation by many genes. Further confirmation of these universal stress responsive genes to irradiation effects may reveal us more detailed mechanisms and potential applications.

**Figure 10:**
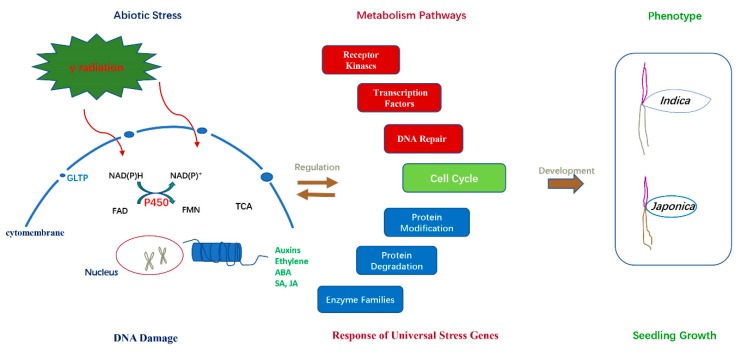
Schematic model for the regulation mechanism in rice plant cells’ response to gamma radiation.

In summary, when plant cells are subjected to gamma radiation, they will initiate the DNA repair mechanism, protein and hormone metabolism, enzyme families and transcription factors pathways, etc. However, the differential expression patterns of universal stress responsive genes confer plants different molecular function and phenotypical difference in *indica* and *japonica* rice varieties (Figure 10).

## 4. Materials and Methods

### 4.1. Plant Materials

Totally 12 rice varieties were used in this study, including seven *japonica*: Nipponbare (NPB), Huaidao5, Yanjing11, Wuyunjing24, Xudao3, Yangjing4038, Yangjing4227 and five *indica*: Yangdao6 (YD6), R1310, R527, R838, Huazhan. NPB and YD6 were selected for RNA sequencing analysis.

### 4.2. Material Treatments and Survey Methods

Dry seeds were exposed to gamma radiation at these dosages: 0, 50, 100, 150, 250, 300, 400, and 500 Gy. After irradiation these seeds were soaked in tap water for four days and germinated at 32 °C for 8 hrs and then at room temperature (25 °C) on the 5th day. The seed germination potential is enumerated on the 2nd day post-germination and the seed germination rate on the 5th day. Morphological investigation features of shoot and root was on the 10th day. About 3000 rice seeds from each treatment were sowed in nursery field for seedling survival test. The seedling germination rate in field was counted on the 7th day and the survival rate on the 30th day. Calculation equations are as follows: seed germination potential = (number of seeds germinated within 2 days/total number of seeds) × 100%, seed germination rate = (number of germinated seeds within 6 days/total number of seeds) × 100%, seedling emergence rate = (number of emerged seeds/number of sown seeds) × 100%, seedling survival rate = (number of seedlings survived on the 30th day/number of sown seeds) × 100%. Three biological replicates were investigated and each biological replicate contained one hundred seeds, statistically significant are labeled as * (*p* ≤ 0.05) or ** (*p* ≤ 0.01).

### 4.3. Image Scanning of Root and Shoot

Root and shoot lengths were investigated by scanning samples at 12 h interval for seven consecutive days. After irradiation, seeds were placed in petri dish and kept at 30 °C in an incubator. Root and shoot were removed from fifty seedlings on the 7th day and washed them with deionized water before placing them in a colorless, transparent pan filled with deionized water. Tweezers were used to adjust the position of root or shoot to avoid overlap. A scanner (EPSON10000XL, Epson America Inc., Long Beach, CA, USA) was used to generate image before subject to analyze by software WinRhizo Pro 2004a (WR, Canada Reagent Instruments Inc., Québec, Canada). Morphological features include length, surface area, average diameter and volume. Treatment has at least three biological replicates and each biological replicate contains fifty seedlings.

### 4.4. Comet Assay

Nuclei were prepared from shoot and root separately. Approximately 0.1 g of shoot and root tissues was chopped quickly in 200 ul of pre-chilled 1× PBS. The extraction mixture was filtered via 200 μm nylon mesh after incubation on ice 8 min. Approximately 30 μL of resulting suspension of nuclei was mixed with 70 μL of 1% low-melting-point agarose and spread on the microscope slide, which was pre-coated with 1% normal-melting-point agarose. After solidification for 20 min at room temperature, the nuclei were subjected to lysis by incubation for 80 min at 4 °C in a high salt solution (2.5 M NaCl, 100 mM EDTA, 10 mM Tris–HCl pH 7.5) and followed by neutralization at 4 °C using 1xTBE solution three times for 5 min each. The neutralized nuclei were electrophoresed in a pre-chilled 1xTBE solution for 30 min at room temperature. After electrophoresis, the slides were kept to clear the starch grains from the gels in 1% of Triton X-100 for 10 min and dehydrate in 75% and 90% ethanol for 5 min respectively and air drying. The nuclei were stained with propidium iodide (Sigma, Cat. # p4170, Zwijndrecht, Netherlands), and images captured by using a fluorescence microscope (Olympus DP80, Olympus Corporation, Shinjuku, Tokyo, Japan) equipped with a CCD camera. DNA damage signals were quantified using the comet assay software project (CASP, Trevigen Inc., Gaithersburg, MD, USA).

### 4.5. Sequencing Sample Preparation and mRNA Library Construction

For RNA sequencing sample preparation, uniformly germinated seeds of NPB and YD6 were prepared before the irradiation. On the 3rd day after germination, the plants were exposed to γ-irradiation of 50 Gy (treatment), and 0 Gy (control). A total of twenty-four fresh samples containing two cultivars (YD6 and NPB), two tissues (shoot and root), two treatments (0 Gy and 50 Gy) and three biological replicates were prepared. All samples were collected immediately after the irradiation.

Total RNA was extracted using the mirVana miRNA Isolation Kit (Ambion, Thermo Fisher Scientific, Inc., Waltham, MA, USA) following the manufacturer’s protocol. RNA integrity was evaluated using the Agilent 2100 Bioanalyzer (Agilent Technologies, Santa Clara, CA, USA). The samples with RNA Integrity Number (RIN) ≥ 7 were subjected to the subsequent analysis. The libraries were constructed using TruSeq Stranded mRNA LTSample Prep Kit (Illumina, San Diego, CA, USA) according to the manufacturer’s instructions. Then these libraries were sequenced on the Illumina sequencing platform (Illumina HiSeq X Ten, Illumina, Inc., San Diego, CA, USA) and 150 bp paired-end reads were generated. Total RNA was isolated from 8 samples with three biological replicates, including root and shoot. These three biological replicates from the NPB and YD6, respectively. NG0R, NG0S indicate the control (0 Gy) of root and shoot in NPB, respectively. NG5R, NG5S indicate the treatment (50 Gy) of root and shoot in NPB, respectively. YG0R, YG0S indicate the control of root and shoot in YD6, respectively. YG5R, YG5S indicate the treatment of root and shoot in YD6, respectively.

### 4.6. Functional Annotation of Transcripts and Mapman Analysis

FPKM value of each gene was calculated using Cufflinks algorithm [20], and the read counts of each gene were obtained by htseq-count [47]. DEGs were identified using the DESeq (2012) functions estimate Scale Factors and nbinom Test. The FPKM value of each gene from the treatment (50 Gy) versus the control (0 Gy) was calculated according to the following criteria: adjusted *p*-value ≤ 0.05 and fold change ≥ 2 or fold change ≤ 0.5 was set as the threshold for significantly differential expression. The genes with FPKM values that fit the above criteria were considered for further analysis. Hierarchical cluster analysis of DEGs was performed to explore transcripts expression pattern. AgriGO was used to perform GO enrichment analysis (http://systemsbiology.cau.edu.cn/agriGOv2/, last accessed on: 31 August 2019). GO enrichment and KEGG pathway analysis were respectively performed using R based on the hypergeometric distribution.

Mapman software was used to analyze DEGs through the metabolism, large enzymes families, cellular response and transcription overview (http://mapman.gabip.org, last accessed on: 31 August 2019). For classifying the DEGs (561 genes in radiation response and 2691 genes in varietal effect), the RGAP gene model of 552 genes in radiation response and 2350 genes in varietal effect were uploaded to the MapMan tool. The remaining genes were not assigned to MapMan terms due to unknown functions.

### 4.7. Validation of RNA-Seq by Qutantitative RT-PCR Analysis

Total rice RNA was extracted using an RNA Prep Pure Plant kit (Tiangen Co., Beijing, China) and chromosomal DNA contamination was removed using DNAase following the manufacturer’s instructions. DEGs were selected based on their possible functions and expression patterns in RNA-Seq. Primer sequences (Supplementary Table S11) were designed using the Beacon Designer 7 software. The rice actin gene (LOC_Os03g13170) was used as reference gene (primer pair Actin). RT-qPCR was performed in BIO-RAD CFX96 Real-Time PCR System. To generate cDNA for real-time quantitative RT-PCR, 1μg of total RNA and oligo (dT) 20 primer were used for reverse transcription (SuperScript™ III RT kit, Invitrogen, CA, USA). Real-time qRT-PCR was conducted under the following conditions: 94 °C/5 min; 44 cycles of 95 °C/30 s, 57 °C/30 s (the annealing temperature for 30 s) 72 °C/30 s; and 72 °C/10 min using SYBR green as a probe (Molecular Probe, Invitrogen). Quantification analysis was performed relative to a standard curve according to the cycle threshold values generated. Fold change relative to control level was determined by the 2^−ΔΔCt^ method [48]. All PCR quantifications were performed in triplicate technical repeats for each sample.

### 4.8. Co-Expression Network Analysis for Construction of Modules

WGCNA (v1.29) package in R was used to construct the co-expression networks [21]. From the total of 55987 genes, 27074 genes with an averaged FPKM ≥ 1 were used for the WGCNA unsigned co-expression network analysis. Through testing the independence and the average connectivity degree of different modules with different power value, the appropriate power value was determined as eight. The modules were obtained using the automatic network construction function with default parameters in WGCNA software package. The top ten genes with maximum intra-modular connectivity were considered as “highly connected gene” (hub gene) [49]. The candidate hub gene network was visualized by the Cytoscape (version 3.6.1) [50].

## 5. Conclusions

This is a pioneer study to reveal the transcriptomic changes in response to irradiation between *japonica* and *indica* rice subspecies. The development of shoots and roots is co-regulating the final rice seedling growth after irradiation, and the stability of agronomic traits in shoot part is more important for survival than that in root part. The core genes related to DNA repair and replication and cell proliferation are similarly regulated by irradiation, however the universal stress responsive genes are in contrasting differential response pattern between the *japonica* and *indica* rice subspecies. We identified specific modules that are associated with the phenotypic traits and candidate hub genes for each module. In conclusion, these results shed light on the regulatory mechanism and the difference between *japonica* and *indica* in response to irradiation stress in rice.

## Figures and Tables

**Table 1:** Candidate hub genes associated with the scale of length and surface area.

Gene Name	Description	K_ME_ Value	Group
**Magenta module with positive correlation**			
LOC_Os08g44750	Auxin-induced protein 5NG4, putative, expressed	0.97	
LOC_Os02g03270	AT hook motif domain containing protein, expressed	0.94	
LOC_Os09g25620	CPuORF8 - conserved peptide uORF-containing transcript, expressed	0.92	
LOC_Os07g05380	ATPase BadF/BadG/BcrA/BcrD type, putative, expressed	0.91	
LOC_Os06g25950	Expressed protein	0.90	
LOC_Os10g40530	LTPL146 - Protease inhibitor/seed storage/LTP family protein precursor, expressed	0.90	
LOC_Os02g25020	DNA binding protein, putative, expressed	0.87	
LOC_Os07g48030	Peroxidase precursor, putative, expressed	0.82	
LOC_Os02g40070	AP2-like ethylene-responsive transcription factor PLETHORA 2, putative, expressed	0.80	
LOC_Os10g08780	Expressed protein	−0.70	
**Royalblue module with negative correlation**			
LOC_Os11g19880	O-methyltransferase, putative, expressed	0.99	DEGs
LOC_Os02g24642	Photosystem II reaction center protein K precursor, putative, expressed	0.96	
LOC_Os06g38890	Transposon protein, putative, unclassified, expressed	0.93	
LOC_Os04g45720	Aldehyde dehydrogenase, putative, expressed	0.92	
LOC_Os02g21550	Flavonol synthase/flavanone 3-hydroxylase, putative, expressed	0.91	DEGs
LOC_Os12g25450	O-methyltransferase, putative, expressed	0.91	
LOC_Os01g27260	Glutathione S-transferase, putative, expressed	0.90	
LOC_Os12g13340	Expressed protein	0.85	DEGs
LOC_Os08g17510	Sulfotransferase domain containing protein, expressed	−0.71	
LOC_Os02g04755	Transposon protein, putative, unclassified, expressed	−0.73	

**Table 2:** Candidate hub genes associated with the scale of average diameter.

Gene Name	Description	K_ME_ Value	Group
**Turquoise module with positive correlation**			
LOC_Os01g03380	BBTI6 - Bowman-Birk type bran trypsin inhibitor precursor, putative, expressed	0.94	
LOC_Os07g44480	Peroxidase, putative, expressed	0.93	
LOC_Os06g08690	Leucine-rich repeat receptor protein kinase EXS precursor, putative, expressed	0.92	
LOC_Os02g37580	Fimbrin-like protein 2, putative, expressed	0.92	
LOC_Os05g10780	Aminotransferase, classes I and II, domain containing protein, expressed	0.91	
LOC_Os10g07040	Chalcone synthase, putative, expressed	0.90	
LOC_Os05g49380	OsDegp9—Putative Deg protease homologue, expressed	0.90	
LOC_Os08g43760	Carrier, putative, expressed	0.87	DEGs
LOC_Os08g43890	Carrier, putative, expressed	0.86	
LOC_Os03g25370	Peroxidase precursor, putative, expressed	0.84	
**Black module with negative correlation**			
LOC_Os01g43650	WRKY11, expressed	0.96	
LOC_Os01g09080	WRKY107, expressed	0.95	DEGs
LOC_Os01g64020	Transcription factor, putative, expressed	0.92	
LOC_Os02g52850	Receptor-like protein kinase like protein, putative, expressed	0.91	
LOC_Os02g11020	Cytochrome P450 72A1, putative, expressed	0.91	
LOC_Os02g03710	UP-9A, putative, expressed	0.81	
LOC_Os01g71094	Basic 7S globulin 2 precursors, putative, expressed	−0.62	
LOC_Os02g32060	Hydrolase, NUDIX family, domain containing protein, expressed	−0.66	
LOC_Os02g10880	UDP-glucoronosyl and UDP-glucosyl transferase domain containing protein, expressed	−0.67	
LOC_Os01g48710	Heavy metal-associated domain containing protein, expressed	−0.79	

**Table 3:** Candidate hub genes associated with the size of volume.

Gene Name	Description	K_ME_ Value	Group
**Pink module with positive correlation**			
LOC_Os04g49270	tRNA-splicing endonuclease positive effector-related, putative, expressed	0.97	DEGs
LOC_Os07g34190	Chalcone and stilbene synthases, putative, expressed	0.97	DEGs
LOC_Os01g65100	Peptide transporter, putative, expressed	0.95	DEGs
LOC_Os07g15460	Metal transporter Nramp6, putative, expressed	0.94	DEGs
LOC_Os08g15322	Cytochrome b559 subunit alpha, putative, expressed	0.92	
LOC_Os09g23540	Dehydrogenase, putative, expressed	0.91	DEGs
LOC_Os08g34310	ZOS8-05 - C2H2 zinc finger protein, expressed	0.91	
LOC_Os01g72370	Helix-loop-helix DNA-binding domain containing protein, expressed	0.90	DEGs
LOC_Os11g45290	Retrotransposon protein, putative, unclassified, expressed	0.89	DEGs
LOC_Os11g39230	Retrotransposon protein, putative, unclassified, expressed	0.89	DEGs
**Purple module with negative correlation**			
LOC_Os02g05970	Phytosulfokine receptor precursor, putative, expressed	0.98	DEGs
LOC_Os03g11490	Expressed protein	0.95	DEGs
LOC_Os10g24690	Expressed protein	0.95	DEGs
LOC_Os06g47120	Expressed protein	0.95	DEGs
LOC_Os01g29390	Expressed protein	0.94	DEGs
LOC_Os10g07150	Transposon protein, putative, unclassified, expressed	0.93	DEGs
LOC_Os11g36200	Receptor-like protein kinase 2 precursor, putative, expressed	0.93	DEGs
LOC_Os06g51360	LysM domain containing protein, putative, expressed	0.90	
LOC_Os09g02000	Expressed protein	0.87	DEGs
LOC_Os04g43680	MYB family transcription factor, putative, expressed	−0.83	

## Supplementary Materials

Supplementary materials can be found at https://www.mdpi.com/1422-0067/20/18/4358/s1.

## Abbreviations

CASP	comet assay software project
DSBs	double strand breaks
DEGs	differentially expressed genes
MCM	mini-chromosome maintenance protein
FPKM	fragments per kilo-base per million reads
GO	gene ontology
KEGG	Kyoto encyclopedia of genes and genomes
RNA-Seq	RNA-sequencing
qRT-PCR	quantitative real-time polymerase chain reaction
UDP	uridine diphosphate
