# Supplementary material for: Global Transcriptome and Co-Expression Network Analysis Reveal Contrasting Response of Japonica and Indica Rice Cultivar to γ Radiation
Source: Int J Mol Sci. 2019 Sep 5;20(18):4358. doi: 10.3390/ijms20184358 (PMC6769861; doi:10.3390/ijms20184358)
Supplement: Supplementary file 1 [file ijms-20-04358-s001.zip › Supplement files/Figure S1-14+Table S1-18/Figure Supplement Legends +Table Supplement Legends.pdf]

Figure S1. Correlation between shoot and root lengths at different irradiation dosages (0Gy, 50Gy, 100Gy, 150Gy, 250Gy, 300Gy, 400Gy and 500Gy) in *japonica* varieties. Each biological replicate contains one hundred seeds and three biological replicates. ** represents significant difference at $p \leq 0.01$.

Figure S2. Correlation between shoot and root length at different irradiation dosages (0Gy, 50Gy, 100Gy, 150Gy, 250Gy, 300Gy, 400Gy and 500Gy) in *indica* varieties. Each biological replicate contains one hundred seeds and three biological replicates. ** represents significant difference at $p \leq 0.01$.

Figure S3. Correlation between the length, surface area, average diameter and volume in shoot and root changes in *japonica* rice NPB and *indica* rice YD6. Each biological replicate contains one hundred seeds and three biological replicates. * and ** represent significant difference at $p \leq 0.05$ and 0.01, respectively.

Figure S4. Global profiling of RNA sequencing results (**A**) Principal Component Analysis (PCA) of the RNA-seq data; (**B**) Cluster analysis of the samples

Figure S5. Overall analysis of gene expression levels. (**A, C**) Clean reads to the reference genome using tophat in NPB (**A**) and YD6 (**C**). (**B, D**) Reads distribution in different components in NPB (**B**) and YD6 (**D**)

Figure S6. Functional analysis of differentially expressed genes (DEGs). (**A, B**) GO enrichment analysis in top 30 entries of irradiation response (**A**) and cultivar effect (**B**). (**C, D**) KEGG enrichment analysis in top 20 entries of irradiation effect (**C**) and cultivar effect (**D**).

Figure S7. 82 DEGs of opposite response to irradiation in the 561 common DEGs. The red color represents the up-regulated change and the blue color represents the down-regulated change.

Figure S8. 47 DEGs of opposite response to variety in the 2691 common DEGs. The red color represents the up-regulated change and the blue color represents the down-regulated change.

Figure S9. Expression of certain DEGs in response to variety. (**A**) AP2 domain-related genes; (**B**), B3 DNA binding domain-related genes; (**C**), Calmodulin binding protein-related genes; (**D**)

glutathione S-transferase-related genes; (**E**) heavy metal associated/transport/detoxification-related genes; (**F**) NBS-LRR disease resistance protein-related genes; (**G**) Cytochrome P450-related genes; (**H**) Glycosyl hydrolase-related genes; (**I**) Transponson protein-related genes-related genes; (**J**) UDP-glucoronosyl/UDP-glucosyl transferase-related genes; (**K**) Zinc finger protein-related genes; (**L**) Leucine rich repeat protein-related genes

Figure S10. DEGs profiling and MapMan analysis to root and shoot in tissue response. (**A**) Venn diagrams of differentially expressed genes (DEGs) in tissue. (**B**) GO enrichment in top 10 items in DEGs between root and shoot in tissue response. (**C**) Results of mapping genes to Metabolism. (**D**) Large enzyme families overview. (**E**) Cellular responses overview. (**F**) Transcription. Red boxes, up-regulated genes; green boxes, down-regulated genes.

Figure S11. The detailed expression data for other modules. Top half of the figure represents the heatmap showing the FPKM of each gene and lower part of the figure represents the eigengene expression profile in 24 samples.

Figure S12. Co-expression network analysis of average diameter related modules. (**A, B**) Gene co-expression networks of positive correlation turquoise module (**A**) and negative correlation black module (**B**) visualized using Cytoscape software platform. The size of the circle and the depth of the color indicate the degree of connectivity of the gene. (**C, D**) The correlation networks of top 20 nodes in turquoise module (**C**) and black module (**D**). The depth of the color represents the number of associated node genes, respectively

Figure S13. Co-expression network analysis of volume related modules. (**A, B)** Gene co-expression networks of positive correlation pink module (**A**) and negative correlation purple module (**B**) visualized using Cytoscape software platform. The size of the circle and the depth of the color indicate the degree of connectivity of the gene. (**C, D**) The correlation networks of top 20 nodes in pink module (**C**) and purple module (**D**). The depth of the color represents the number of associated node genes, respectively

Figure S14. Expression level of hub genes. (**A**) Turquoise module and black module; (**B**) Pink module and purple module.

Table S1. Statistics of reads and bases in all samples in the RNA sequencing

Table S2. Primers used for qRT-PCR in this study

Table S3. 561 common DEGs in response to irradiation

Table S4. 2961 common DEGs in response to variety

Table S5. GO enrichments of induced genes in YD6/NPB in response to irradiation

Table S6. GO enrichments of reduced genes in YD6/NPB in response to irradiation

Table S7. GO enrichments of opposite change genes in YD6/NPB in response to irradiation

Table S8. GO enrichments of induced genes in YD6/NPB in response to variety

Table S9. GO enrichments of reduced genes in YD6/NPB in response to variety

Table S10. GO enrichments of opposite change genes in YD6/NPB in response to variety

Table S11. 3673 common DEGs in response to tissue effect

Table S12. GO enrichments of DEGs in tissue response to irradiation

Table S13. GO enrichments in magenta module

Table S14. GO enrichments in royalblue module

Table S15. GO enrichments in turquoise module

Table S16. GO enrichments in black module

Table S17. GO enrichments in pink module

Table S18. GO enrichments in purple module
