# Supplementary figures and images for: Global Transcriptome and Co-Expression Network Analysis Reveal Contrasting Response of Japonica and Indica Rice Cultivar to γ Radiation
Source: Int J Mol Sci. 2019 Sep 5;20(18):4358. doi: 10.3390/ijms20184358 (PMC6769861; doi:10.3390/ijms20184358)

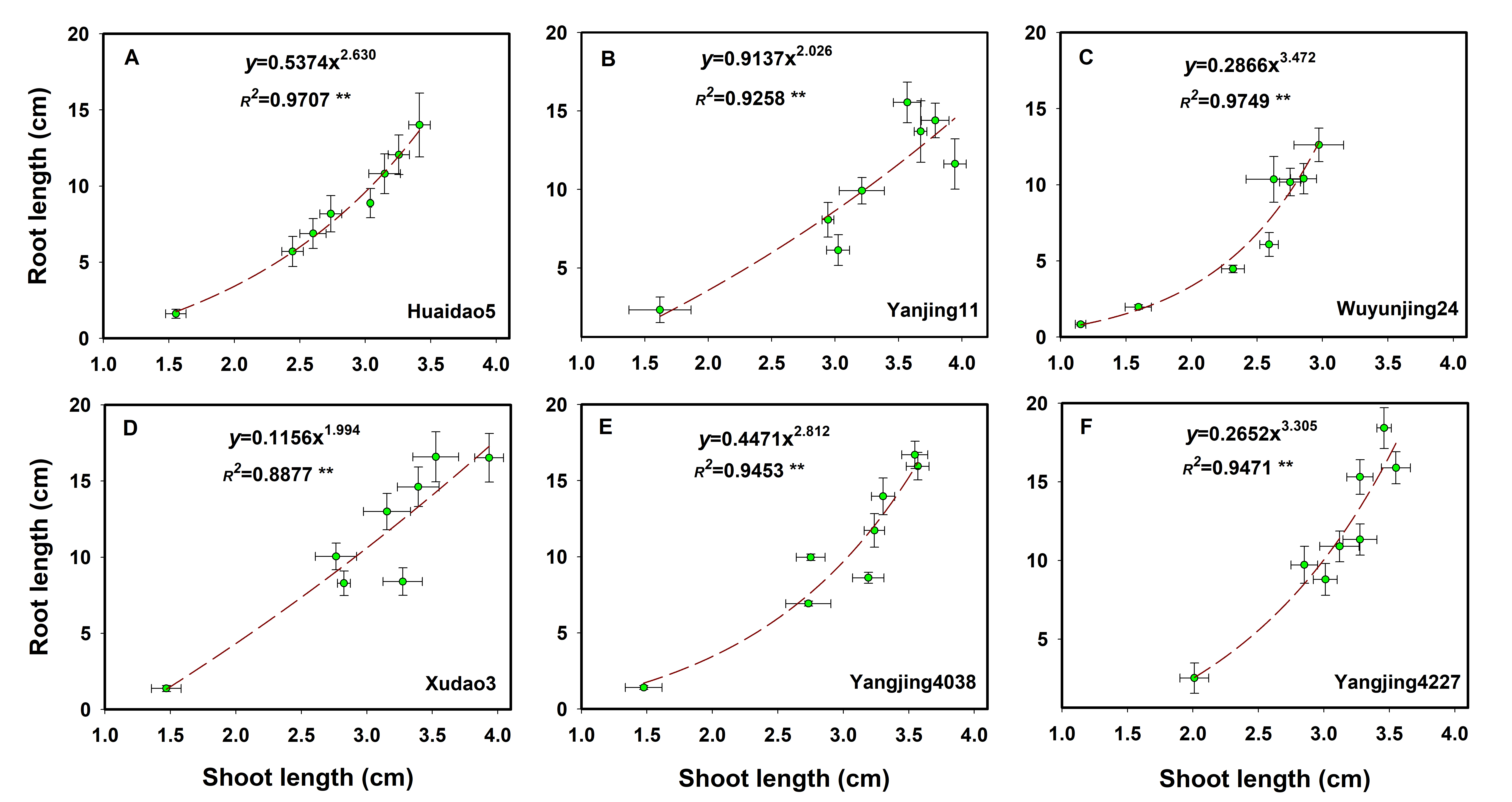

Supplement: Supplementary file 1 [file ijms-20-04358-s001.zip › Supplement files/Figure S1-14+Table S1-18/Figure S1.JPG]

A

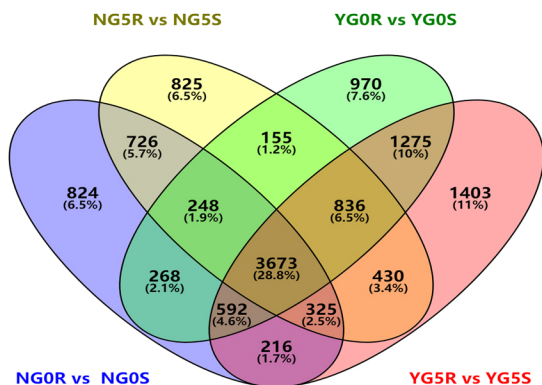

B

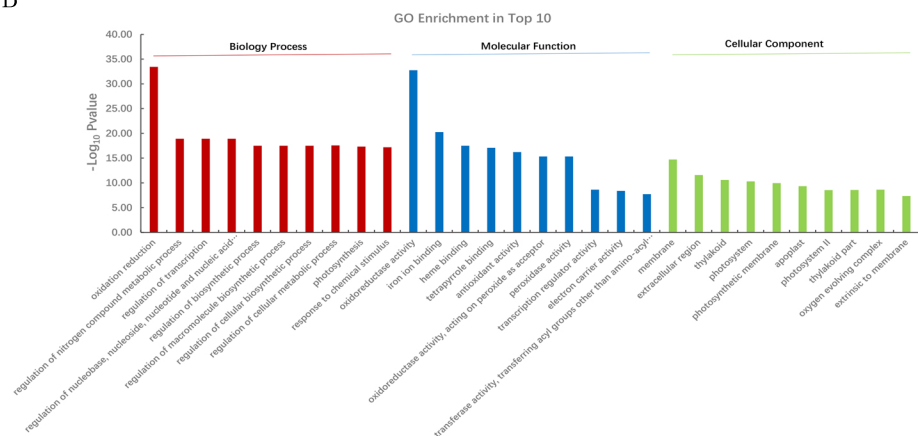

C

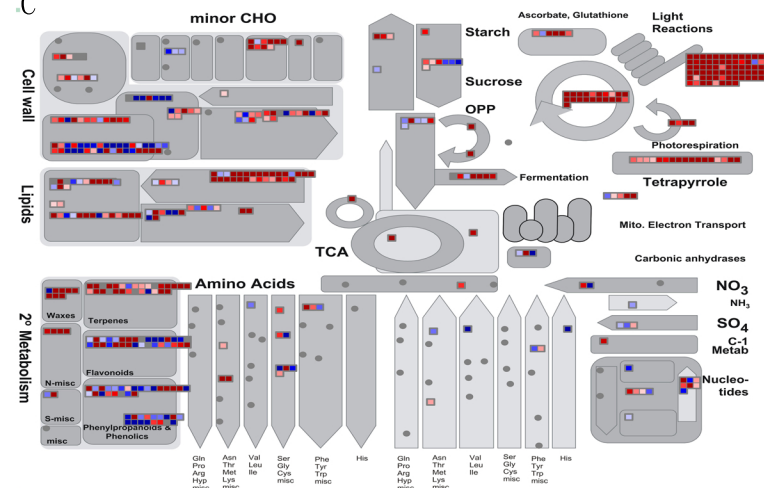

D

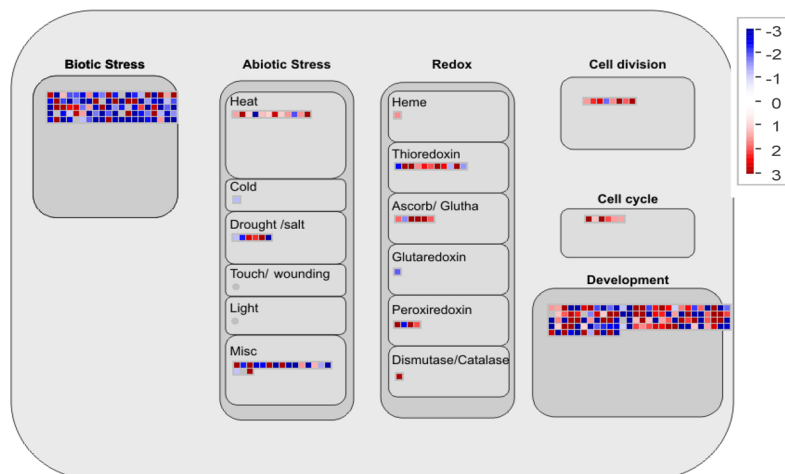

E

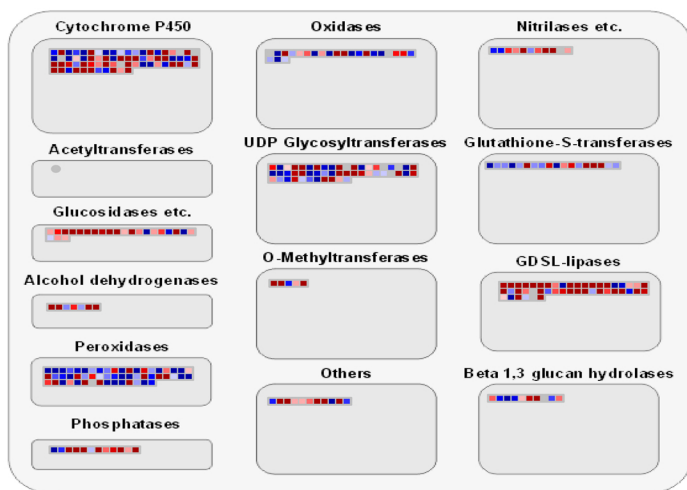

F

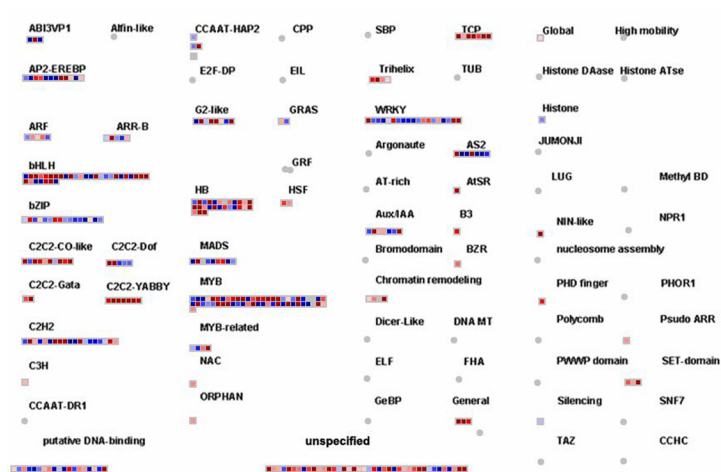

Supplement: Supplementary file 1 [file ijms-20-04358-s001.zip › Supplement files/Figure S1-14+Table S1-18/Figure S10.pdf]

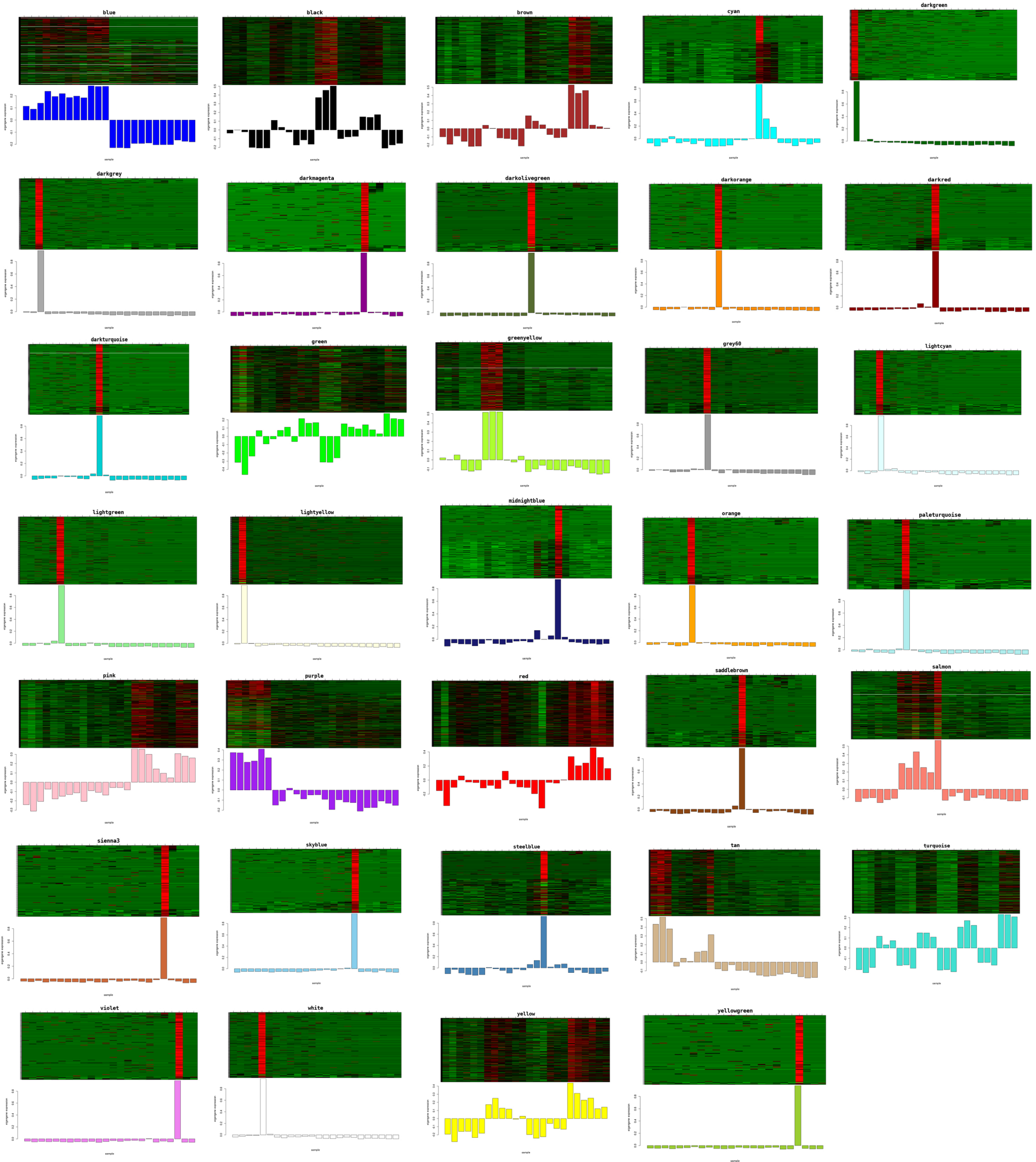

Supplement: Supplementary file 1 [file ijms-20-04358-s001.zip › Supplement files/Figure S1-14+Table S1-18/Figure S11.pdf]

A

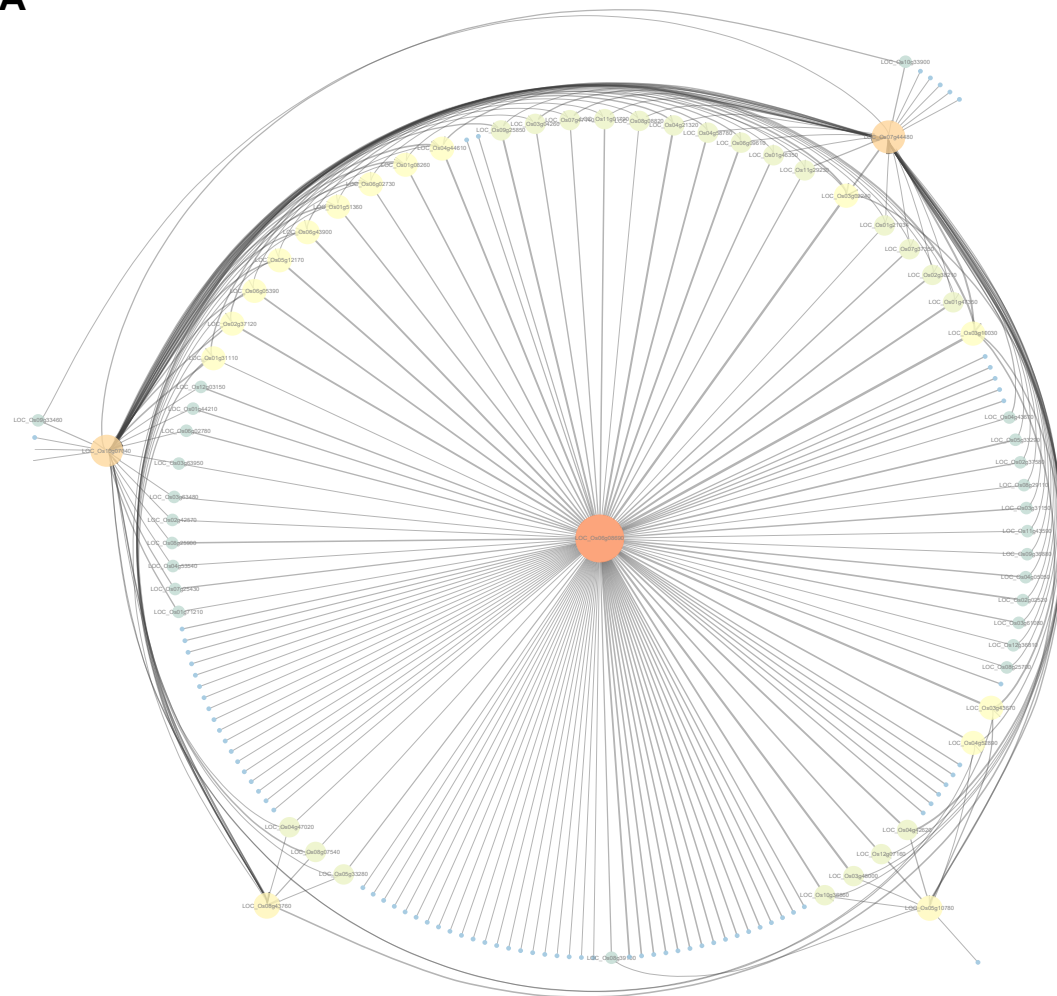

B

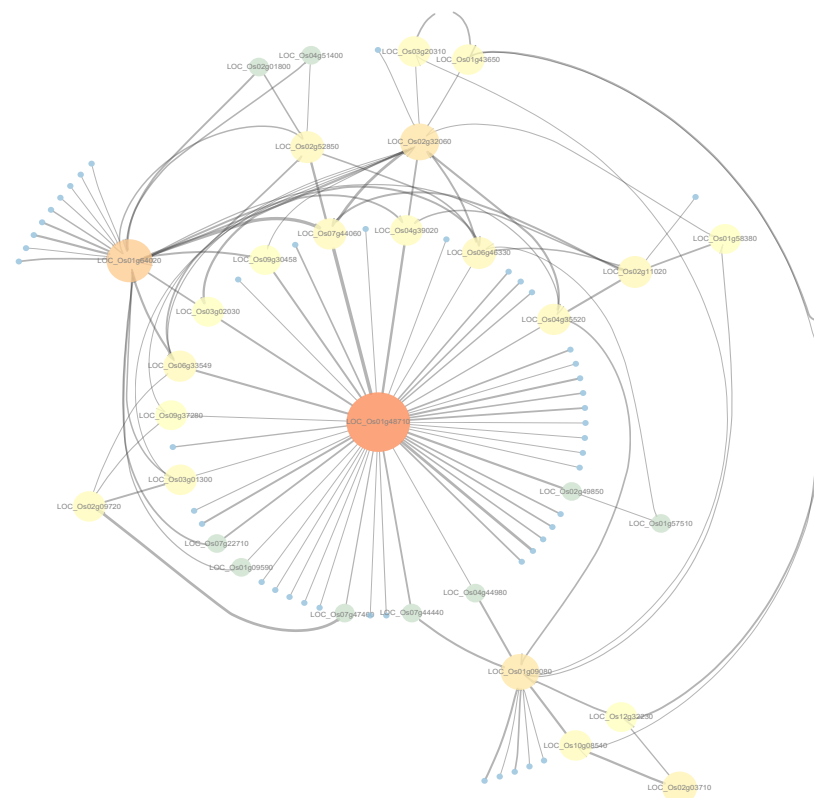

C

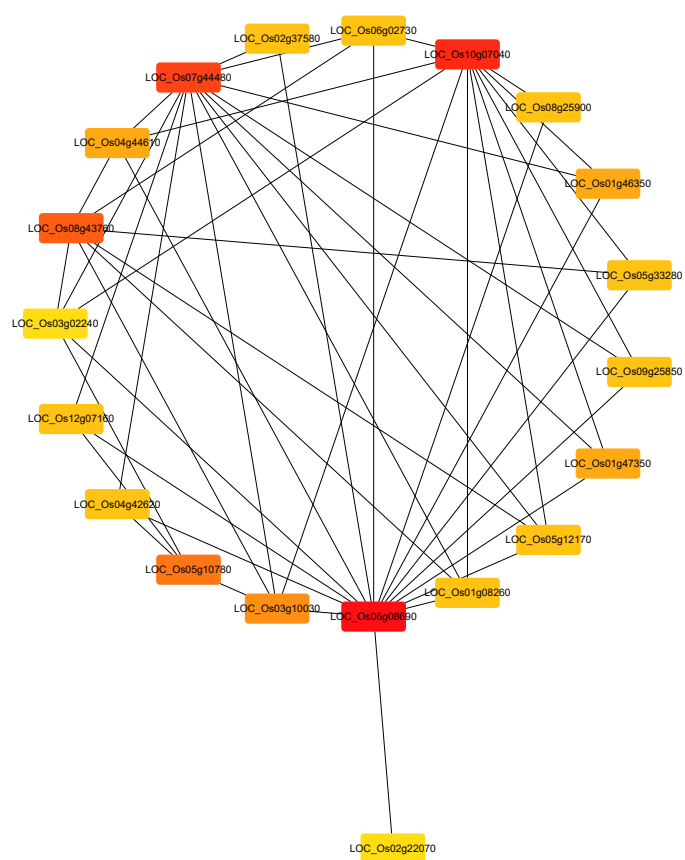

D

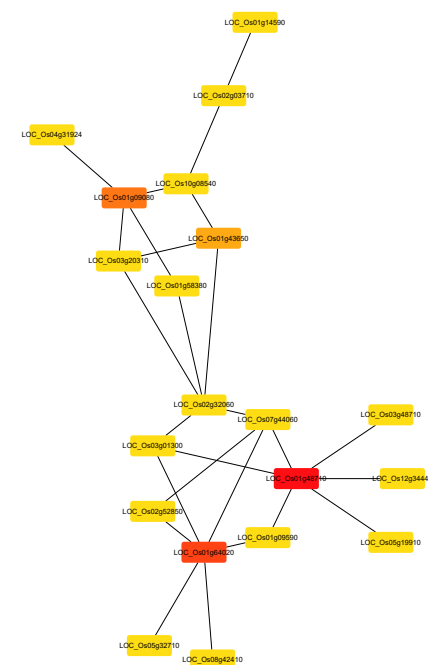

Supplement: Supplementary file 1 [file ijms-20-04358-s001.zip › Supplement files/Figure S1-14+Table S1-18/Figure S12.pdf]

A

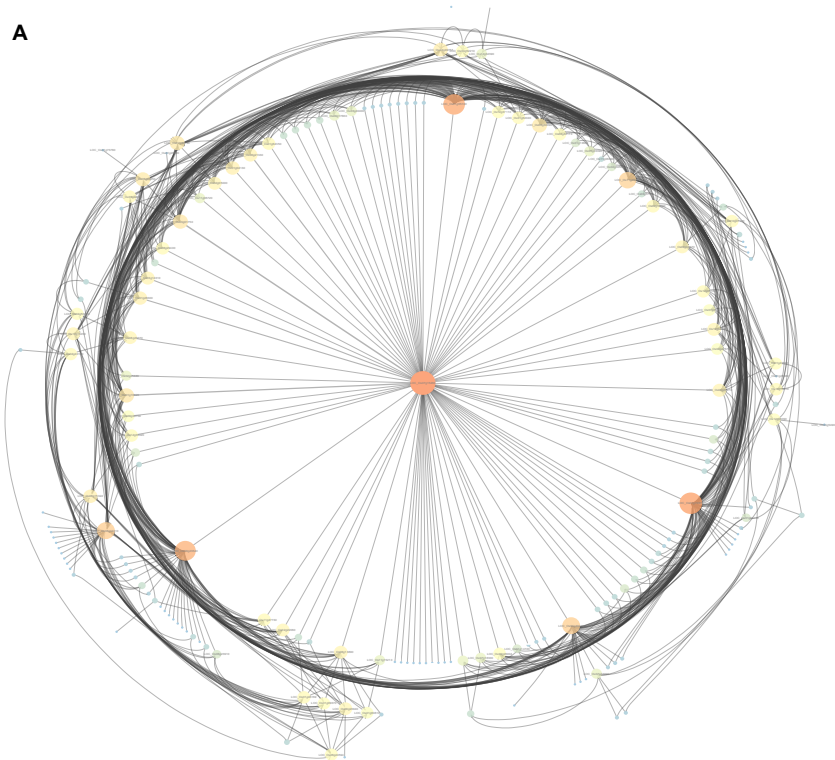

B

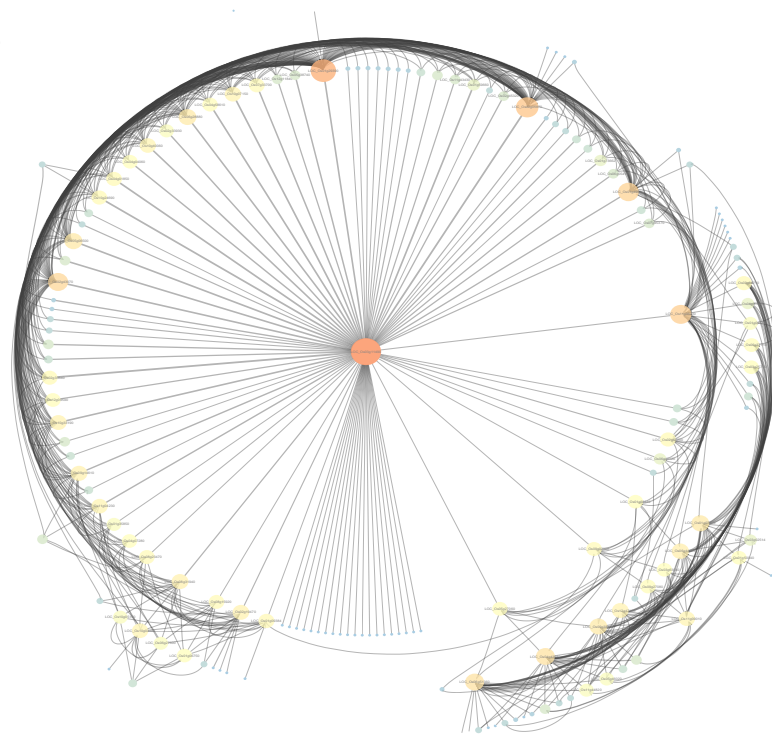

C

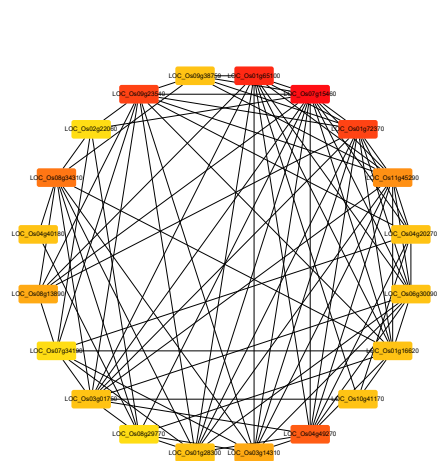

Top 20 nodes

D

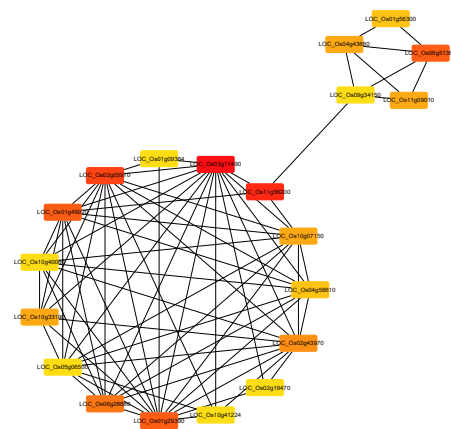

Top 20 nodes

Supplement: Supplementary file 1 [file ijms-20-04358-s001.zip › Supplement files/Figure S1-14+Table S1-18/Figure S13.pdf]

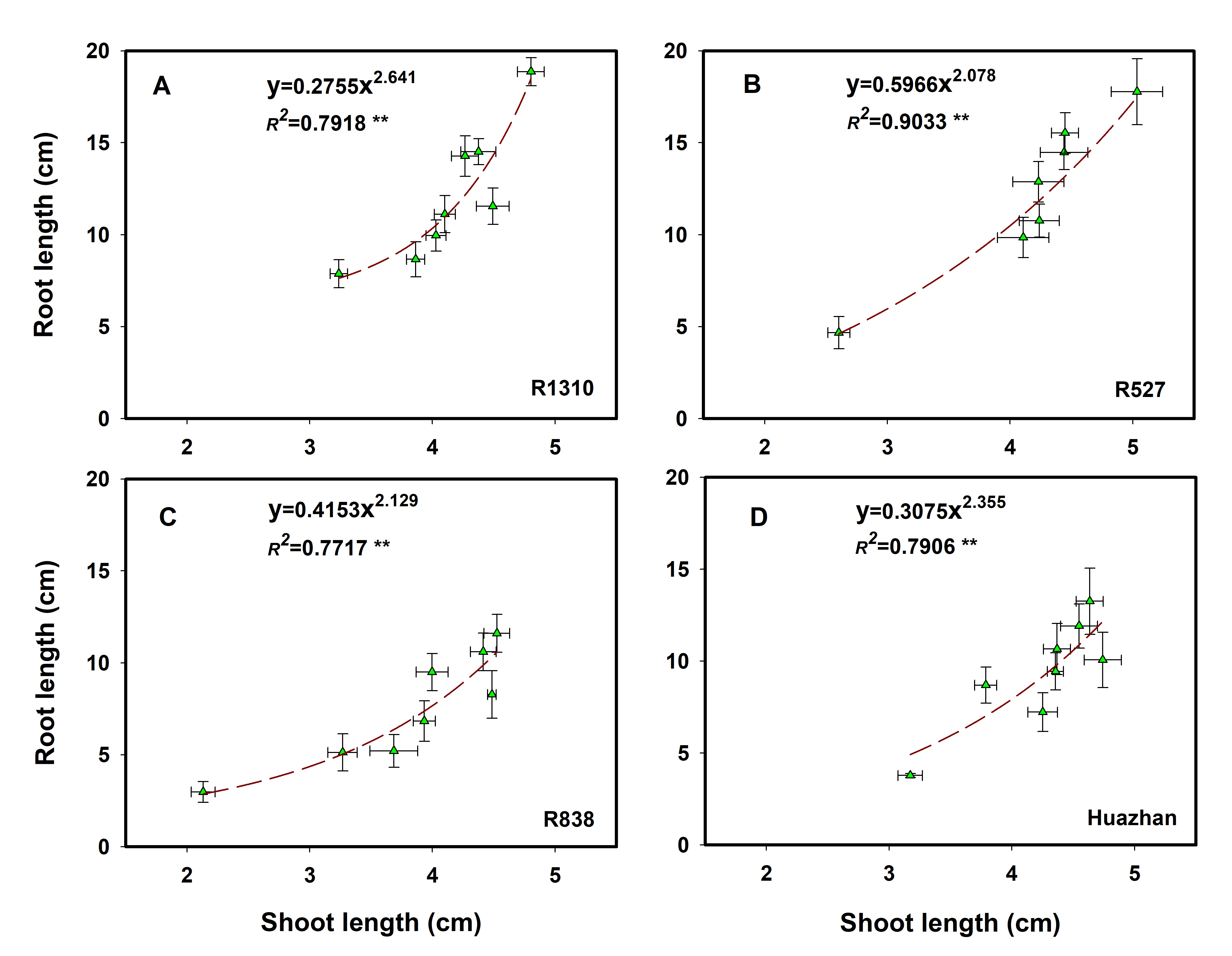

Supplement: Supplementary file 1 [file ijms-20-04358-s001.zip › Supplement files/Figure S1-14+Table S1-18/Figure S2.JPG]

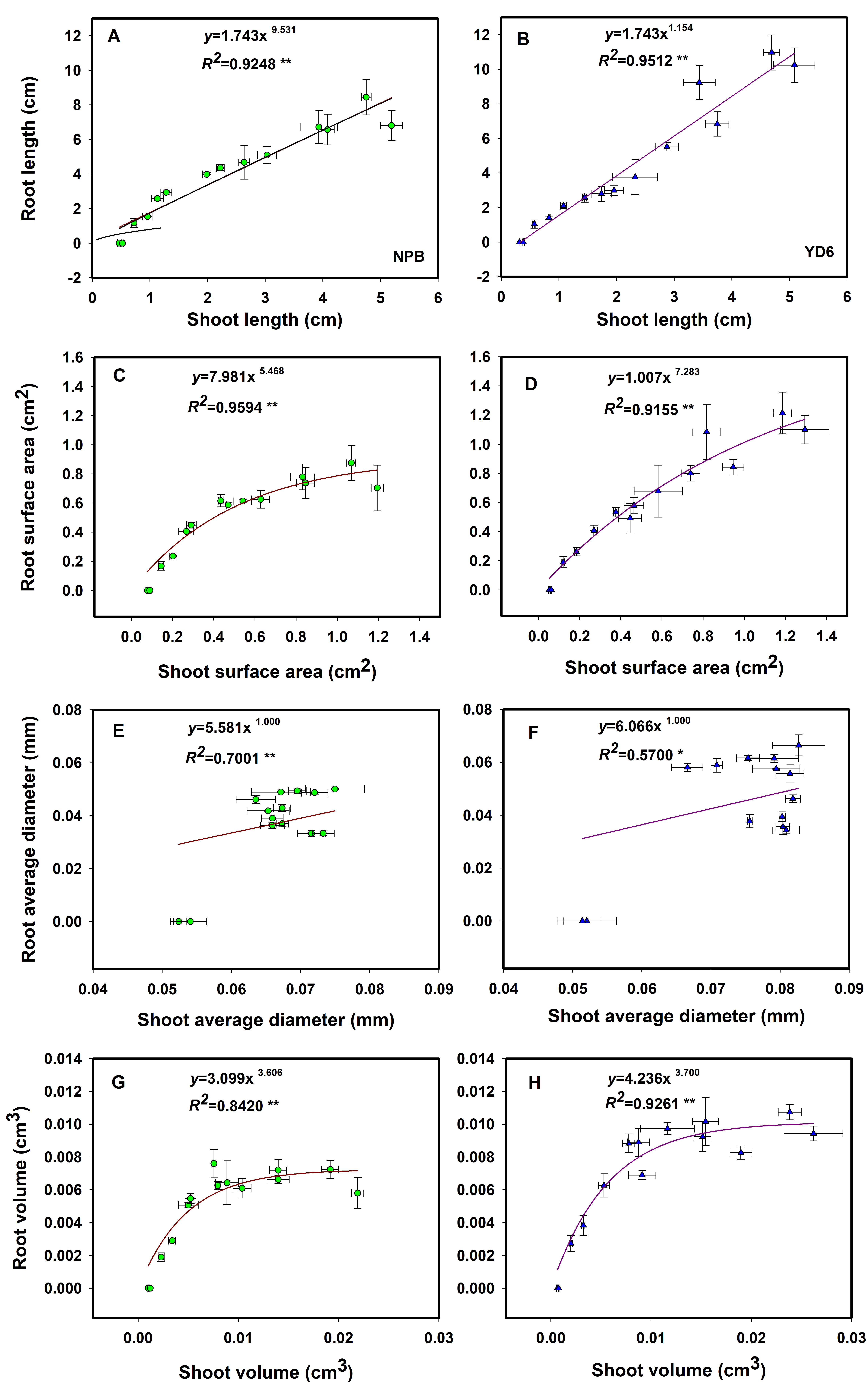

Supplement: Supplementary file 1 [file ijms-20-04358-s001.zip › Supplement files/Figure S1-14+Table S1-18/Figure S3.JPG]

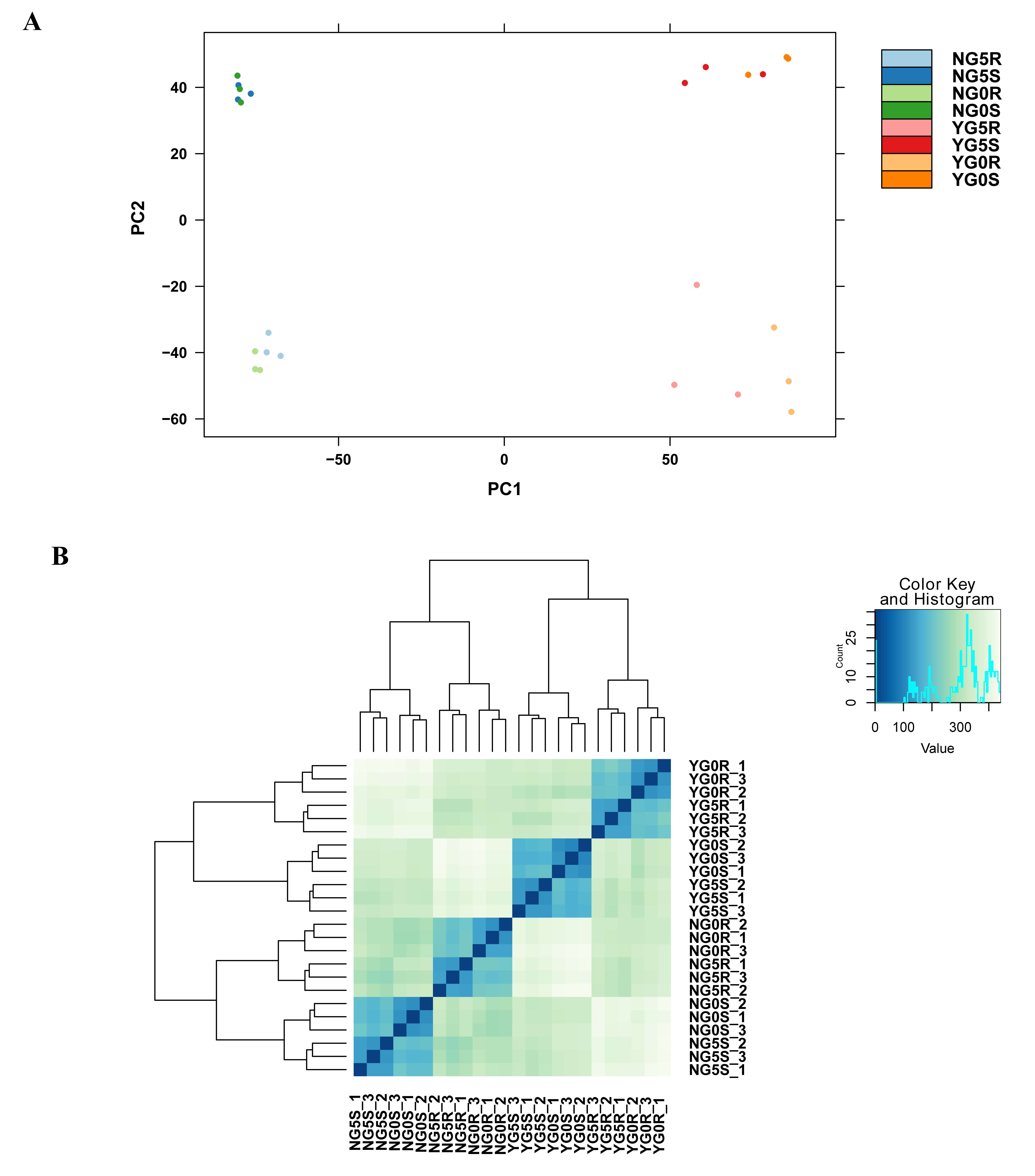

Supplement: Supplementary file 1 [file ijms-20-04358-s001.zip › Supplement files/Figure S1-14+Table S1-18/Figure S4.tif]

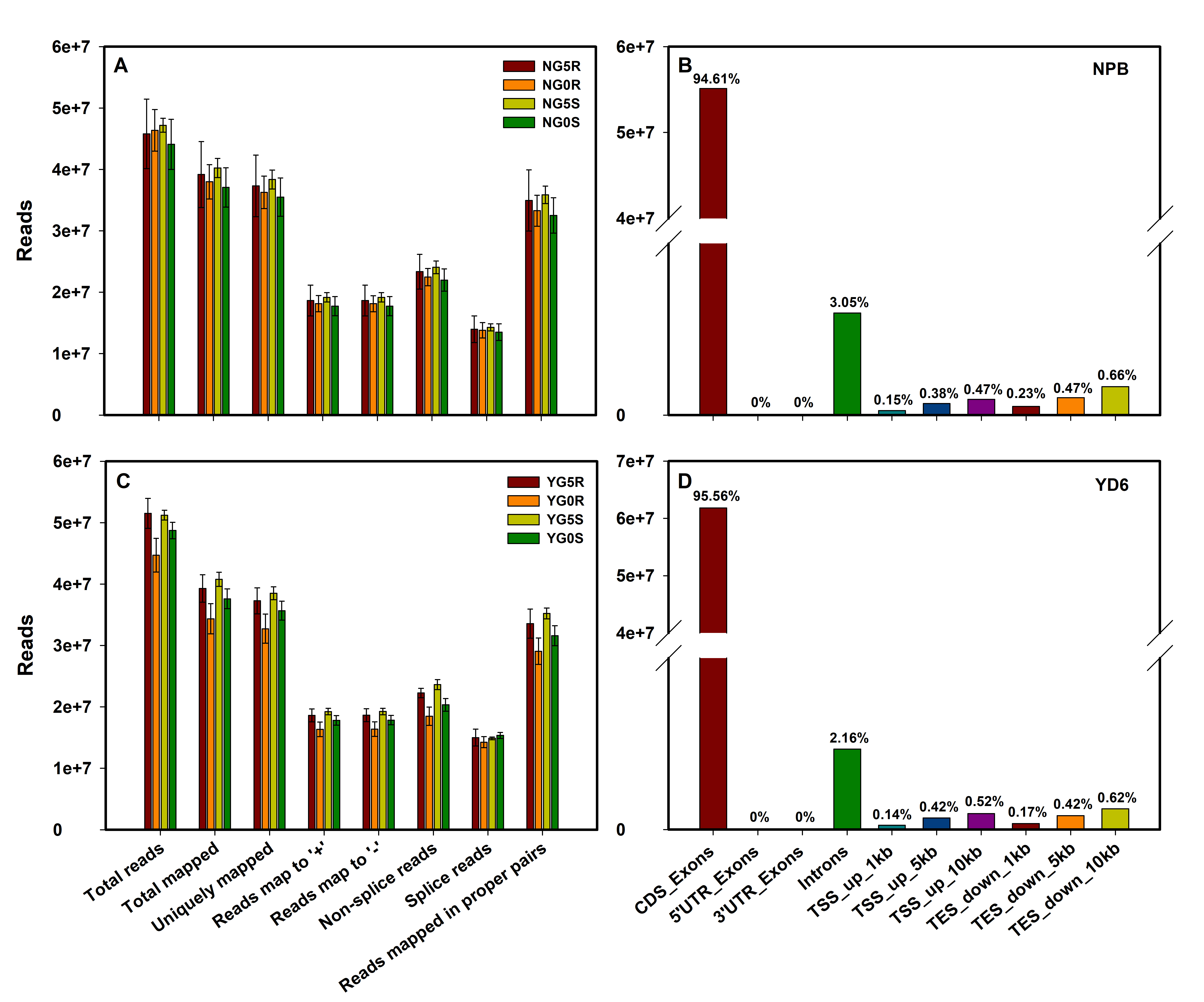

Supplement: Supplementary file 1 [file ijms-20-04358-s001.zip › Supplement files/Figure S1-14+Table S1-18/Figure S5.JPG]

A

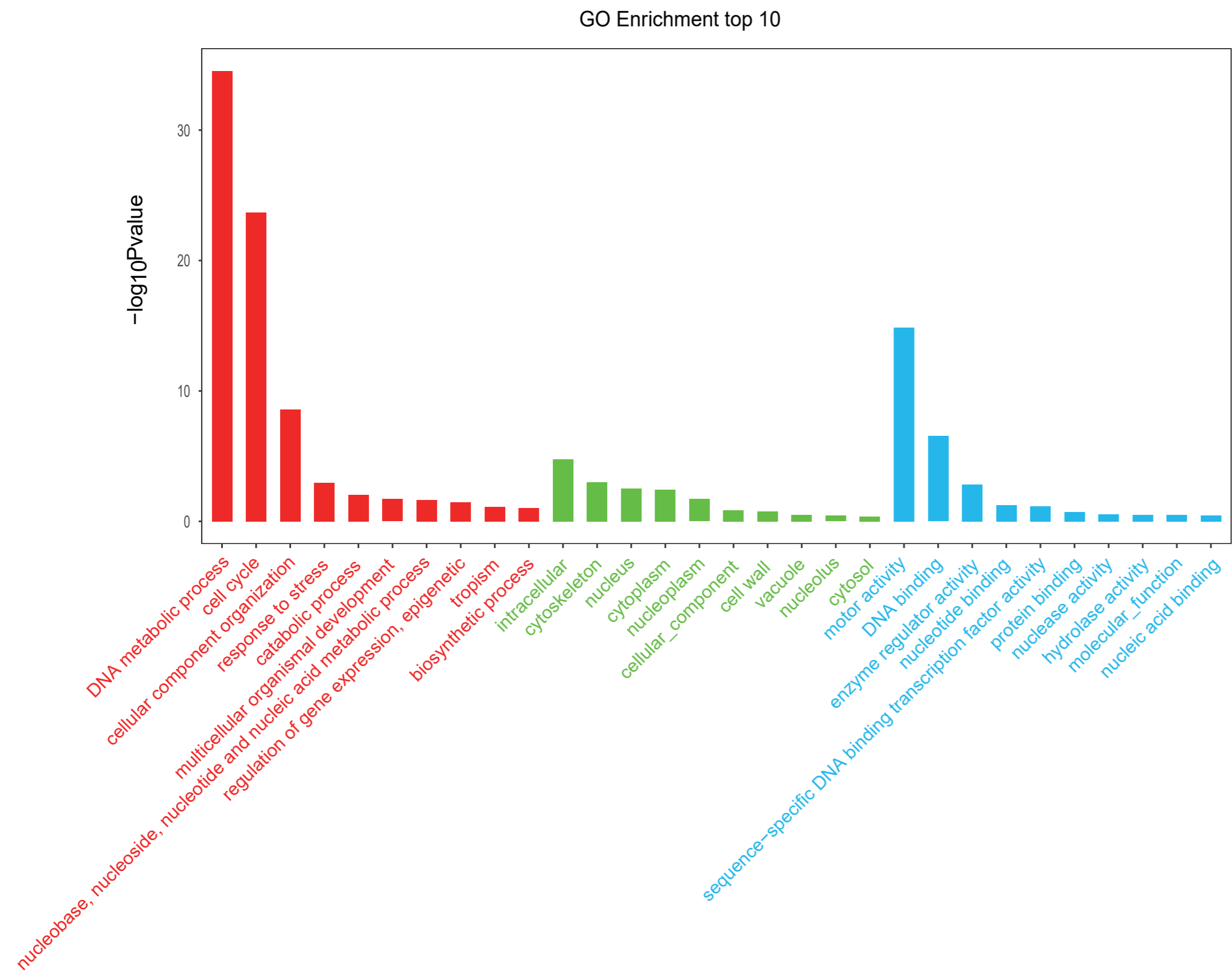

B

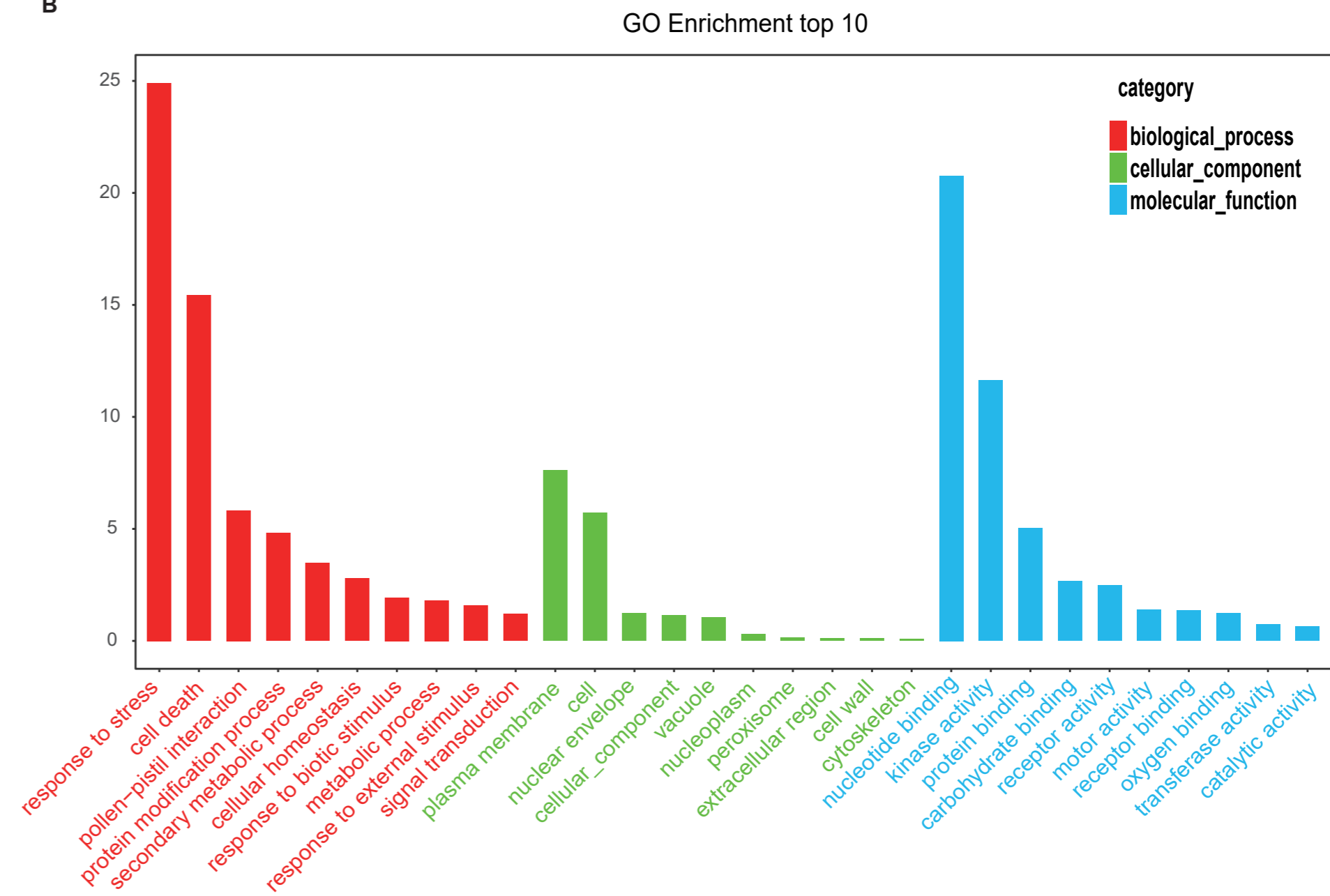

C

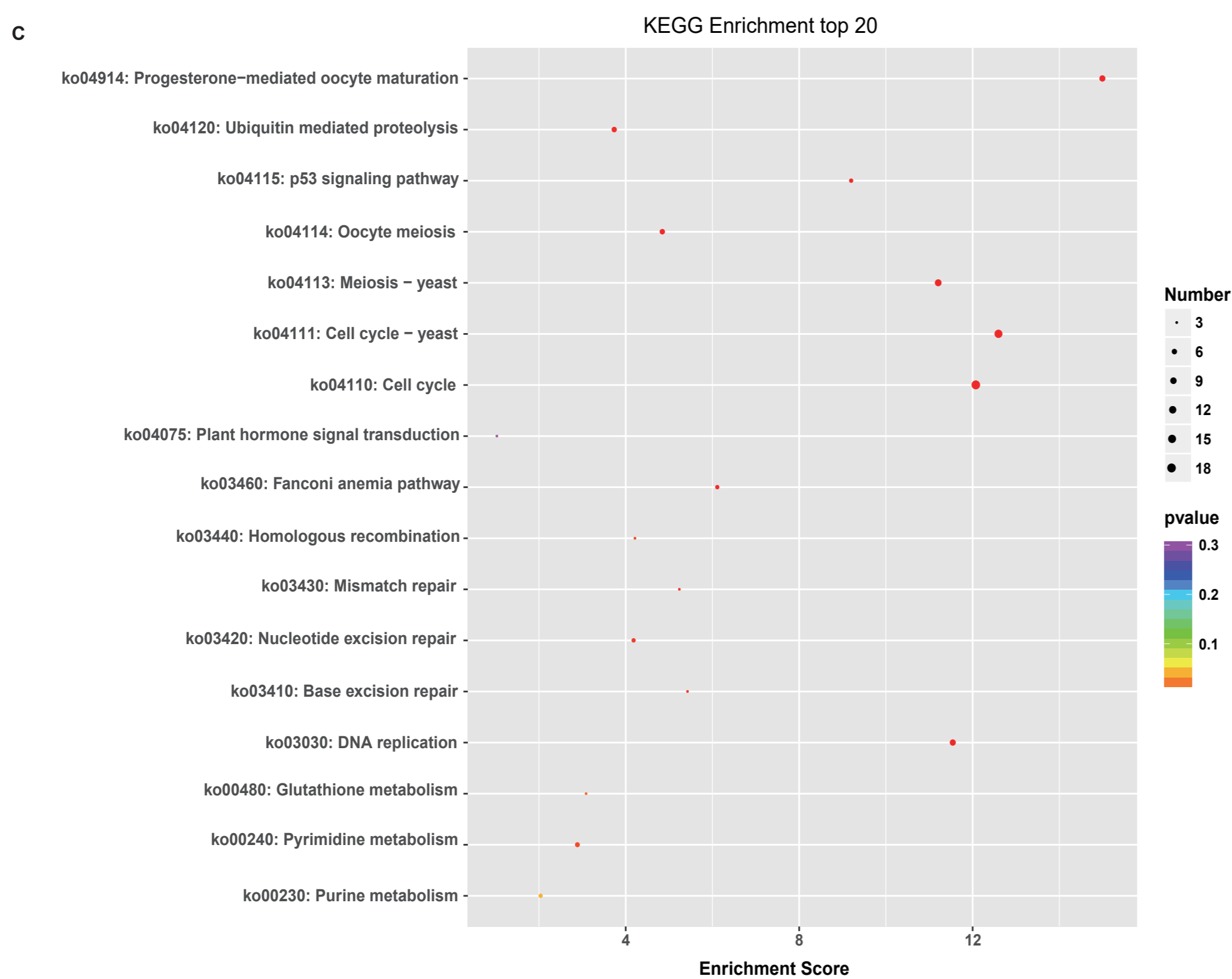

D

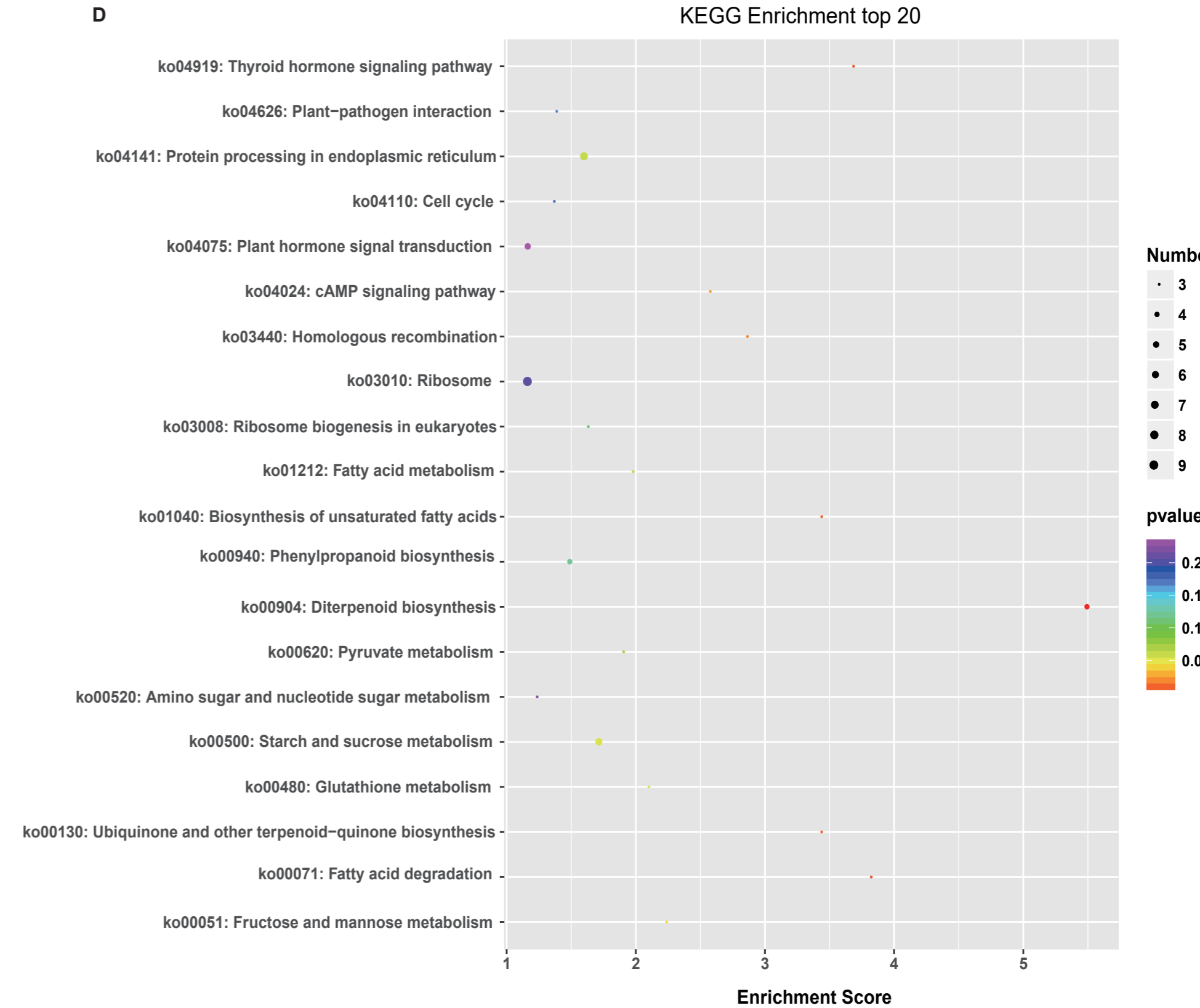

Supplement: Supplementary file 1 [file ijms-20-04358-s001.zip › Supplement files/Figure S1-14+Table S1-18/Figure S6.pdf]

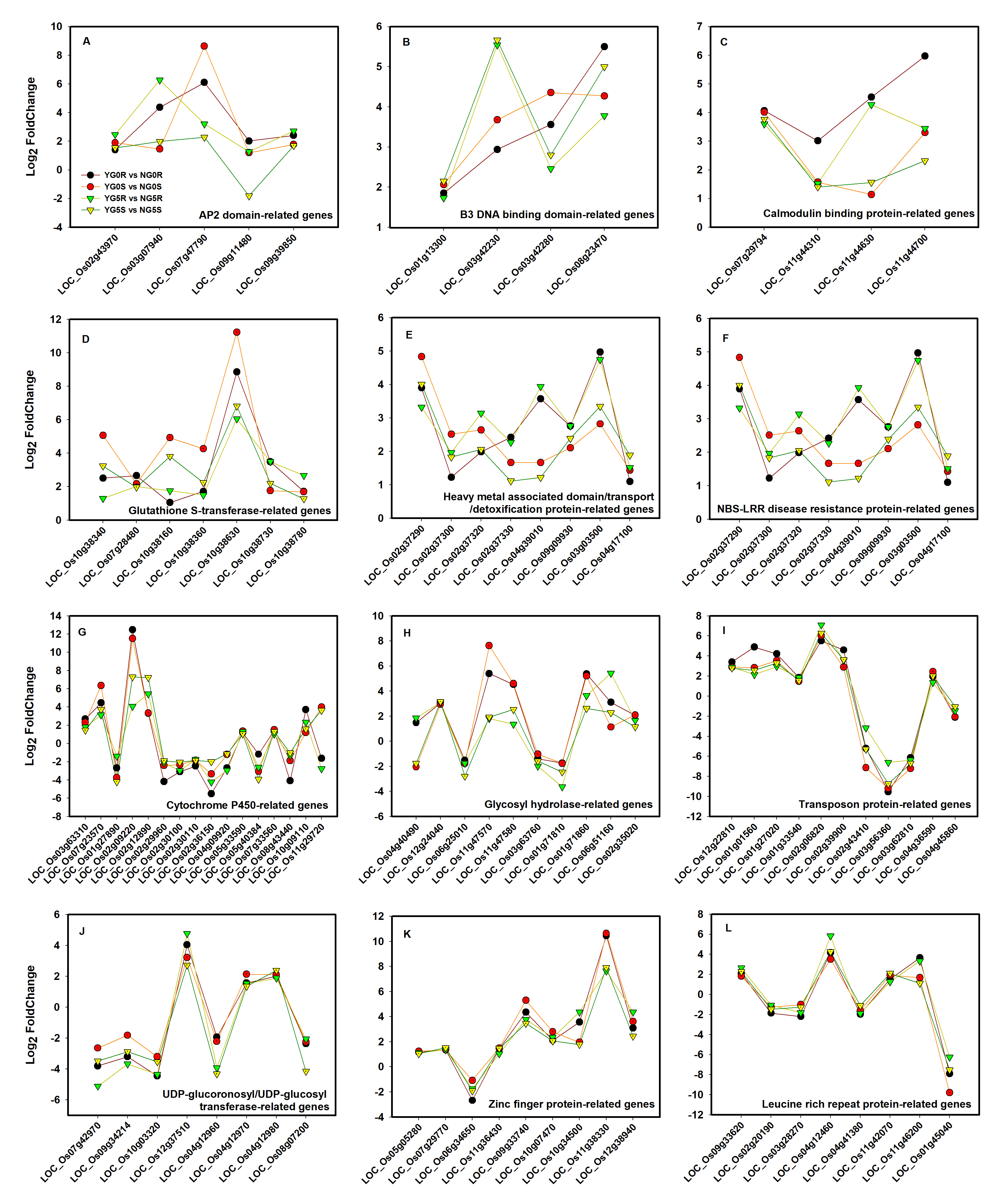

Supplement: Supplementary file 1 [file ijms-20-04358-s001.zip › Supplement files/Figure S1-14+Table S1-18/Figure S9.JPG]
